# Decoding microbiome responses to quarantine potato wart disease: first insights into suppression and biocontrol by full-length 16S rRNA gene profiling and functional prediction

**DOI:** 10.3389/fpls.2025.1707759

**Published:** 2026-02-03

**Authors:** Ishraq Akbar, Yichao Shi, Bart T. L. H. van de Vossenberg, Theo A. J. van der Lee, Lang Yao, Xiang Li, Jiacheng Chuan, Linda E. Jewell, Hai D. T. Nguyen, Wen Chen

**Affiliations:** 1 Ottawa Research & Development Centre, Agriculture & Agri-Food Canada (AAFC), Ottawa, ON, Canada; 2 Department of Biology, University of Ottawa, Ottawa, ON, Canada; 3 Netherlands Institute for Vectors, Invasive Plants and Plant Health, National Plant Protection Organization, Netherlands Food and Product Safety Authority, Wageningen, Netherlands; 4 Biointeractions and Plant Health, Wageningen University & Research, Wageningen, Netherlands; 5 The Ottawa Laboratory (Carling), Canadian Food Inspection Agency (CFIA), Ottawa, ON, Canada; 6 The Charlottetown Laboratory, Canadian Food Inspection Agency (CFIA), Charlottetown, PE, Canada; 7 St. John’s Research and Development Centre, Agriculture & Agri-Food Canada, St. John’s, NL, Canada

**Keywords:** potato wart, microbiome, nanopore sequencing, plant growth promoting bacteria (PGPB), *Synchytrium endobioticum*

## Abstract

**Introduction:**

*Synchytrium endobioticum*, the biotrophic pathogen causing potato wart, poses persistent challenges due to its long-term soil survival and quarantine status. Biological control agents (BCAs) offer a promising avenue for sustainable management, yet the ecological context of wart-associated microbiomes remains unexplored.

**Methods:**

We present the first comprehensive microbiome characterization of the potato wart disease system using full-length 16S rRNA gene Nanopore sequencing across bioassay-grown warts, field-collected wart tissues, diseased tare soils, and long-term descheduled (wart-free) soils. Whole genome amplification (WGA) enabled profiling of low-biomass samples, albeit with compositional shifts towards dominant taxa.

**Results and discussion:**

Microbiome compositional structure differed significantly across sieving fractions, host genotypes, and compartments (wart vs. tare soil). Wart microbiomes were enriched in *Pseudomonas trivialis* and *Bacillus atrophaeus*, taxa potentially involved in pathogen-specific suppression. Tare soils harbored transitional microbiomes shaped by host proximity, enriched with *Bacillus* species that may offer both generalist and targeted BCA activity. Descheduled soils under long-term nonhost crop rotations harbored broad-spectrum BCAs contributing to environmental sensing and nutrient requisition. Functional prediction suggested enrichment of xenobiotic degradation and chitin metabolism pathways in diseased soils, primarily associated with *Bacillus, Pseudomonas*, and *Paenibacillus*. Network analysis indicated fragile yet densely connected communities in diseased soils versus modular and stable structures in descheduled systems. Altogether, this study represents a first critical step toward developing biocontrol strategies for *S. endobioticum* by revealing a gradient of biocontrol reservoirs associated with disease pressure and management history. The use of functional prediction and correlation network tools provides essential starting points for hypothesis-driven research into disease suppression and biocontrol in a system with no prior microbiome data, and these findings warrant targeted isolation and *in vitro*/*in planta* validation for BCA development.

## 1 Introduction

Potato wart is a soil-borne disease characterized by the formation of cauliflower-like galls, primarily on stolons and tubers of potato (*Solanum tuberosum* L.) (Hampson, 1993). It is caused by *Synchytrium endobioticum* (Schilberszky) Percival, an obligate biotrophic chytrid fungus, capable of causing up to 100% yield losses and rendering affected tubers unmarketable (van de Vossenberg et al., 2022b). Owing to its severe economic impact and persistence in soil, *S. endobioticum* is classified as a high-risk quarantine pathogen in many potato-producing countries, including Canada and the Netherlands, where it was first identified in the early 20^th^ century (Hampson, 1993; Baayen et al., 2006; Government of Canada, 2012; van de Vossenberg et al., 2022b).

Managing *S. endobioticum* infection remains a formidable challenge due to the pathogen’s ability to produce highly resilient resting spores (winter spores) within hypertrophic wart tissues. Upon tissue decay or mechanical disruption, these spores are released into the surrounding soil, where they can remain viable for several decades (van de Vossenberg et al., 2022b). Once conditions become conducive (typically cool, moist soils), the resting spores germinate to produce motile zoospores capable of initiating new infections (Curtis and Blackman, 1921; EPPO Bulletin, 2017b; van de Vossenberg et al., 2022b). *S. endobioticum* can be disseminated to new locations via infested soil, infected seed tuber material, or contaminated agricultural equipment (van de Vossenberg et al., 2022b), exacerbating the risk of spread and reinforcing the need for vigilant surveillance and containment strategies.

Potato wart disease is primarily managed by strict phytosanitary regulations and the cultivation of resistant potato cultivars (van de Vossenberg et al., 2022b). Long-term descheduling of infested fields demands extensive timelines, sometimes exceeding 20 years (EPPO Bulletin, 2017b). Additional control measures, such as soil fumigation (Monro et al., 1970), chitin-based amendments (Hampson and Coombes, 1995), and fungicide applications (Çakir and Demirci, 2023) have also been investigated. However, chemical treatments pose risks to public and environmental health (Hampson, 1988; Zhang et al., 2024) and have demonstrated limited and inconsistent efficacy against *S. endobioticum* largely due to the extreme durability of its resting spores and the short treatment duration or low number of application cycles in existing studies (Obidiegwu et al., 2014). As a result, these chemical interventions are neither widely recommended nor approved for practical field management of potato wart. Management is further complicated by the presence of over 40 recognized *S. endobioticum* pathotypes globally, with breeding efforts for broad-spectrum resistance facing limited success (Obidiegwu et al., 2014; van de Vossenberg et al., 2022b). For instance, a 2020 outbreak in Stadskanaal, Netherlands, involving the emergence of pathotype 38 (Nevşehir) affected cultivars previously resistant to the (formerly) common pathotypes 1(D1), 2(G1), 6(O1), and 18(T1) (van de Vossenberg et al., 2024).

Given the challenges of managing potato wart, there is growing interest in sustainable alternatives to complement existing control measures. Biological control agents (BCAs) are beneficial microbes or their metabolites that suppress plant pathogens, offering an environmentally friendly strategy by leveraging natural interactions such as competition, antibiosis, and parasitism (Pandit et al., 2022; Chen et al., 2023). Bacterial strains of the genera *Pseudomonas*, *Bacillus*, *Pantoea*, *Flavobacterium*, and *Actinobacteria*, and fungal strains of the genera *Trichoderma*, *Gliocladium*, and *Clonostachys*, have demonstrated biocontrol and plant-growth promoting (PGP) potentials against a wide range of plant diseases (Lahlali et al., 2022). However, to date, no validated BCAs exist for potato wart and they have yet to achieve practical success in field descheduling programs, although *Thiobacillus thiooxydans* was previously tested in a field trial with promising outcome, but was not validated in subsequent repetitions (Obidiegwu et al., 2014).

A promising way for preventive disease control lies in harnessing soil suppressiveness, which is the natural capacity of microbial communities to inhibit pathogen establishment or proliferation (De Corato, 2020; Jayaraman et al., 2021). One key mechanism is soil fungistasis, whereby indigenous microbes prevent fungal spore germination through resource competition or antagonistic activities (Supronienė et al., 2023). Suppressive soils have been documented against some soilborne pathogens, such as *Fusarium oxysporum* (El-kazzaz et al., 2022), *Phytophthora* spp (Ozgonen and Erkilic, 2007), and *Rhizoctonia solani* (Barnett et al., 2006). Moreover, pathogen stress can enrich for beneficial endophytes with plant-growth-promoting and biocontrol traits, while plants themselves shape microbiomes through rhizodeposition and legacy effects (Yang et al., 2020; Hannula et al., 2021). These observations align with the keystone pathogen hypothesis, which posits that even low-abundance pathogens can exert disproportionate effects on host-microbiome interactions and ecological structure (Hajishengallis et al., 2012; Banerjee et al., 2018). Although *S. endobioticum* spores may be present in low quantities in infested soils, their ability to cause disease suggests biologically meaningful interactions that could leave detectable microbiome signatures. However, whether such microbiome-mediated suppressiveness exists or can be engineered against potato wart remains an open question.

Despite increasing interest in microbiome-based disease suppression, research into natural soil fungistasis against potato wart remains limited by several constraints. Quarantine regulations severely restrict access to infected field sites and samples, making large-scale or longitudinal studies difficult. Furthermore, genetic material from *S. endobioticum* is often scarce, limiting the feasibility of *in vitro* and *in vivo* experimentation. Uneven sampling across sample types (such as soil, wart tissue, and bioassay-derived materials), further complicates efforts to unravel complex pathogen-microbiome-plant interactions. Overcoming these barriers is critical for advancing microbiome-informed biocontrol strategies and developing more sustainable approaches to managing potato wart.

This study employed Oxford Nanopore full-length 16S rRNA gene metabarcoding to profile bacterial communities associated with potato wart disease using environmental DNA (eDNA) samples from the Netherlands. Leveraging the improved taxonomic resolution offered by Nanopore long-read sequencing over short-read platforms (Alteio et al., 2021; Zhang et al., 2021; Satam et al., 2023), we investigated how *S. endobioticum* infection alters microbiome composition, potentially selecting for bacterial taxa with biocontrol capabilities. To better identify potential BCAs, functional predictions based on 16S rRNA gene, while inherently inferential, offer valuable ecological insights when direct measurements are not feasible, especially in the context of early-stage studies on quarantine pathogens such as *S. endobioticum*. PICRUSt2 (Douglas et al., 2020), a widely adopted tool in microbial ecology, leverages phylogenetic placement and genome-informed inference to predict functional profiles of microbial communities based on sequenced reference genomes of phylogenetically close taxa. While we recognize the limitations of such predictive tools, their ability to generate functional hypotheses in our current exploratory study is crucial for systems where culturing is constrained by regulatory or logistical barriers (Douglas et al., 2020; Wang et al., 2022). Indeed, recent studies have adopted similar staged approaches using PICRUSt2 to predict microbial functions across diverse ecological systems (Duan et al., 2024; Lee et al., 2025; Tang et al., 2025). For example, Li et al. (2024) revealed contrasts in microbial metabolic activity across soil pore scales, while Vigil et al. (2024) characterized bacterial communities associated with Peruvian macroalgae, both explicitly acknowledging the predictive nature of *PICRUSt2* and the need for experimental validation. In agreement with these studies, we emphasize that our use of PICRUSt2 in this study serves solely to generate testable hypotheses and guide subsequent targeted isolation and functional assays.

We hypothesized that wart disease pressure and management history shape distinct microbiome signatures across field soils, wart tissues, and bioassay systems, revealing core microbial players, fungistasis signals, and legacy effects. To our knowledge, this is the first comprehensive study to characterize microbiome structures and predicted functions associated with potato wart disease using a metagenomics-informed approach. Our findings provide ecological and functional insights into natural suppression and persistence, offering a framework to guide microbiome-based biocontrol strategies for managing *S. endobioticum*.

## 2 Materials and methods

### 2.1 Overall experimental design and research questions

To investigate how *S. endobioticum* infection and disease management influence the potato-associated microbiome, we designed a study incorporating diverse sample types representing different levels of disease pressure, biosecurity constraints, and ecological contexts. We analyzed bacterial communities in four major sample types: naturally infested soils, freshly collected field wart tissues, bioassay-derived wart tissues, and healthy (descheduled) soils. This diversity allowed us to examine microbial variation both within and between environments under different disease statuses.

To enrich for pathogen-associated signals and capture spatial microhabitats, samples were size-fractionated and stratified by origin (field vs. bioassay), helping to distinguish environmental effects from those driven by pathogen or host genotype. The inclusion of wart tissues from Spieckermann bioassays (EPPO Bulletin, 2017b; van de Vossenberg et al., 2019a) was deliberate. These standardized pathogenicity tests, routinely used in quarantine diagnostics, provide a controlled system for reproducible wart formation. They allowed us to investigate wart-associated microbiomes under standardized conditions while also meeting biosecurity requirements that limit direct access to infested field sites.

To overcome low biomass and DNA yield from most of these samples, we employed whole genome amplification (WGA) using multiple displacement amplification, which has been shown to preserve representative taxonomic profiles in low-input samples (Hawkins et al., 2002; Sabina and Leamon, 2015; Ordóñez and Redrejo-Rodríguez, 2023; Ospino et al., 2024; Agyabeng-Dadzie et al., 2025).

The overarching research questions guiding this study are: (i) Can microbiome dynamics across field soils, wart tissues, and bioassay systems reveal signatures of fungistasis, core microbial players, or legacy effects; and (ii) can this knowledge uncover microbial traits or taxa with potential for biocontrol and natural suppression of potato wart disease? To address these objectives within the context of diverse sample types and WGA-enabled low-input profiling, we developed five focused research questions (**Table 1**) that collectively examine microbial shifts, functional potential, biocontrol relevance, and disease suppression mechanisms.

**Table 1:** The five research questions and samples used in this study.

Research questions	DNA samples
1.) Influence of whole genome amplification (WGA) on microbiome recovery from eDNA was assessed using both a mock bacterial community and field-derived environmental samples.	Mock bacterial community (ZymoBIOMICS) • n =3 Newfoundland & Labrador diseased field soil from Avondale (n=14): • Non-WGA DNA (n = 7) • WGA DNA (n = 7)
2.) Differences in microbial community composition between the 25 µm and 75 µm sieving fractions of wart tissues were examined to evaluate size-based microbial partitioning and inform optimal strategies for recovering ecologically relevant taxa.	Netherland wart from Spieckermann bioassay (n=12) • 75 µM sieve fraction (n = 6) • 25 µM sieve fraction (n = 6)
3.) Bacterial communities in field- versus bioassay-derived wart tissues (75 µm fraction) were compared to determine the extent to which bioassay systems replicate the microbiome structure of naturally infected warts.	Netherland wart samples from field & bioassay (75 µM fraction): • Infested field (n =10) • Spieckermann bioassay (n = 6)
4.) Microbial profiles of wart tissues and infested tare soils were analyzed to explore potential transmission pathways and identify reservoirs of candidate biocontrol organisms.	Netherland infested field samples (75 µM fraction): • Wart samples (n = 10) • Tare soil (n = 6)
5.) Bacterial communities in diseased tare soils and healthy (descheduled) soils to identify potential reservoirs of BCAs and PGPB, considering disease pressure and soil legacy effects in the context of potato wart biovigilance.	Netherland soil samples from: • Healthy (descheduled) soil (n = 33, 25 µM fraction) • Diseased (infested) tare soil (n = 6, 75 µM fraction)

### 2.2 Sample requisition from Netherlands

A total of 61 DNA samples were provided by Netherlands’ National Plant Protection Organization (NPPO), encompassing three distinct sample groups (**Supplementary Table S1**). The first group (n = 16) was collected from the Mussel and Onstwedde fields during the 2020 potato wart outbreak in the municipality of Stadskanaal. These fields were infected with *S. endobioticum* pathotype 38 (Nevşehir), and the samples consisted of field wart tissues (n = 10) and associated diseased tare soil (n = 6). Field wart tissues were homogenized using a Grindomix system, followed by sieving through a 75 µm mesh, which retained resting spores of *S. endobioticum*, plant material and debris. Diseased tare soil samples were also processed using a 75 µm sieve. The second group comprised wart tissues (n = 12) obtained from a Spieckermann bioassay, in which potato cultivars Deodara, Saphir, and Talent were inoculated with pathotype 38 (Nevşehir) isolates from either the Mussel or Onstwedde field. Details of the Spieckermann bioassay protocol are provided in **Supplementary Figure S1**. For this group, two size fractions (25 µm and 75 µm) were collected to evaluate microbiome difference based on particle size. The 25 µm fraction retained higher quantity of *S. endobioticum* spores than the 75 µm fraction, confirmed by *S. endobioticum*-specific qPCR assays (Weller et al., 2000; Smith et al., 2014; EPPO Bulletin, 2017b). The third group consisted of healthy (descheduled) soil samples (n = 34) sieved to create a 25 µm fraction as part of a descheduling survey as per EPPO guidelines (EPPO Bulletin, 2017a). Sample processing followed the European and Mediterranean Plant Protection Organization (EPPO) standard protocols for descheduling surveys (EPPO Bulletin, 2017b; van de Vossenberg et al., 2022a), as illustrated in **Supplementary Figure S2**. The 25 µm sieving fraction was used for descheduled soils because it generally retains a higher concentration of *S. endobioticum* resting spores compared to the 75 µm fraction, which was used for potato wart-infested soils. Differences in sieving fraction sizes and unknown field operations under descheduling containment limited the direct comparison of potato wart-infested soils (75 µm fraction) and descheduled soils (25 µm fraction) in the context of microbiomes taxonomic and functional profiling under disease pressures. However, our focus was to evaluate each compartment’s potential as a reservoir of plant growth-promoting bacteria (PGPB) and microbial traits relevant to wart suppression. This conceptual shift allows us to consider the role of both pathogen-driven selection and soil legacy effects in shaping microbiomes with biocontrol potential, especially under restricted access to infested fields.

Netherlands soil samples were confirmed to be infested or free of *S. endobioticum* spores by visual examination and validated by Quantitative PCR (qPCR) assays targeting the pathogen. The qPCR assay (Smith et al., 2014; EPPO Bulletin, 2017b) targets a 143 bp region of the 18S small subunit ribosomal RNA gene (SSU rDNA) using a *S. endobioticum*-specific primer set, Se18S_RTF1 (forward, 5′-CTC TGG TTG AGC TCC ATT TAC-3′) and Se18S_RTR2 (reverse, 5′-CCT ATT CTA TTA TTC CAT GCT GTA-3′), with TaqMan probe Se18S_TM1 (5′- 6-FAM -TAT CCT GGT TCC CCA CAG GCA CTC- BHQ1- 3′) (qPCR reagents listed in **Supplementary Table S2**; qPCR results summarized in **Supplementary Table S3**). Amplification reactions were performed in a real-time PCR thermal cycler (CFX Opus 96, Bio-Rad, CA, USA) under the following conditions: an initial denaturation at 90 °C for 10 minutes, followed by 40 cycles of denaturation at 95 °C for 15 seconds and annealing/extension at 60 °C for 1 minute.

### 2.3 DNA extraction and whole genome amplification

The eDNA samples from The Netherlands as well as Newfoundland & Labrador were extracted using QIAGEN’s DNeasy PowerSoil Pro Kit (QIAGEN, Germany), following the manufacturer’s protocol. Due to low DNA inputs, whole genome amplification (WGA) was used to obtain sufficient input for Nanopore sequencing.

Two amplification kits were tested on a standardized mock community (ZymoBIOMICS D6305, Zymo Research, USA), including the GenomiPhi V3 Ready-To-Go DNA Amplification Kit (Cytiva, USA) and the REPLI-g Single Cell Kit (QIAGEN, Germany) according to the manufacturer’s instructions.

GenomiPhi V3 was selected for further testing on Canadian DNA samples from potato wart-infested fields (n = 7) at the Avondale substation of Agriculture and Agri-Food Canada’s St. John’s Research and Development Center in Newfoundland and Labrador, Canada. The Canadian soil samples were collected from a depth of 0-15 cm using a composite sampling strategy, where three 500 g soil cores were mixed per site. From each composite, two 200 mg replicates were subjected to DNA extraction without prior sieving or drying and subsequently pooled. Although the Avondale site is known to harbor pathotypes 2(G1), 6(O1), and 8(F1), the presence of *S. endobioticum* spores in these specific soil samples was not confirmed.

All WGA-amplified DNA samples were purified the Short Read Eliminator XS Kit (PacBio, USA) to remove DNA fragments less than 5 kb. DNA concentration, quality, and fragment size distribution were assessed on the Agilent TapeStation system (Agilent Technologies, USA), according to the manufacturer’s instructions.

Additionally, a no-template (water) negative control was included throughout WGA, purification, library preparation, and Nanopore sequencing (See Section 2.4) to verify the absence of contamination during all processing steps.

### 2.4 Library preparation and nanopore sequencing

Library preparation for full-length 16S rRNA gene metabarcoding targeted the V1-V9 hypervariable regions (~1.5 kb) using a two-step PCR approach. In the first PCR, amplification was performed using primer pair 27F (5′-TTT CTG TTG GTG CTG ATA TTG CAG AGT TTG ATC MTG GCT CAG-3′) and 1492R (5′-ACT TGC CTG TCG CTC TAT CTT CCG GTT ACC TTG TTA CGA CTT-3′) (dos Santos et al., 2019). The PCR program included initial denaturation at 95 °C for 3 minutes, followed by 40 cycles of 95 °C for 30 seconds, 55 °C for 30 seconds, and 72 °C for 90 seconds, with a final extension at 72 °C for 5 minutes. Amplicon concentration was measured using the Qubit 1× HS dsDNA assay (Thermo Fisher Scientific, USA) on a Qubit 2.0 fluorometer, and product size (~1.5 kb) was confirmed by electrophoresis on a 1% agarose gel at 70 V for 30 minutes.

The second PCR step introduced sample-specific barcodes using the PCR Barcoding Expansion 1–96 Kit (EXP-PBC096; Oxford Nanopore Technologies, UK). Thermal cycling conditions were: initial denaturation at 95 °C for 3 minutes; 15 cycles of 95 °C for 15 seconds, 62 °C for 15 seconds, and 65 °C for 90 seconds; and a final extension at 65 °C for 5 minutes. Barcoded amplicons (~1.6 kb) were verified by gel electrophoresis, and DNA concentrations were re-measured using the Qubit assay (ThermoFisher Scientific, USA).

Barcoded libraries were purified using AMPure XP beads (Beckman Coulter, USA) and visualized on a 1% agarose gel to confirm size and integrity. Samples were pooled in equimolar concentrations based on Qubit quantification. The pooled library was further assessed using the Agilent TapeStation D5000 assay (Agilent Technologies, USA). End repair and clean-up were performed with the NEBNext Ultra II End Repair Kit (New England Biolabs, USA), followed by AMPure XP purification. Adapter ligation was conducted using the Oxford Nanopore SQK-LSK110 and SQK-LSK114 ligation kits, following the manufacturer’s instructions. Final libraries were loaded at 20 fmol and 50 fmol onto MinION flow cells R9.4.1 and R10.4.1, respectively. Sequencing was performed on the MinION platform (Oxford Nanopore Technologies, UK) for 72 hours.

### 2.5 Bioinformatic processing of sequencing data

Raw sequencing data were generated in POD5 format, the native output of the Oxford Nanopore Technologies (ONT) MinION platform, which retains signal-level information for downstream processing. Basecalling was performed using *DORADO* (v0.5.1+a7fb3e3) (Samarakoon et al., 2023) which uses neural network algorithms to decode raw electrical signals into nucleotide sequences, identifying both canonical and modified bases while applying error correction to address the inherent noise of nanopore data. The high-fidelity “sup” (super accuracy) model was used for basecalling: dna_r9.4.1_e8_sup@v3.6 for R9.4.1 flow cells and dna_r10.4.1_e8.2_400bps_sup@v5.0.0 for R10.4.1 flow cells. A single BAM file containing all reads was produced. Demultiplexing into sample-level FASTQ files was also performed using *DORADO*, with adapter trimming disabled at this stage. Read quality was assessed with *FastQC 0.12.1* (Andrews, 2010), and results were summarized across all samples using *MultiQC* 1.25.2 (Ewels et al., 2016). Per-base Phred scores (Ewing and Green, 1998) indicated suboptimal quality, necessitating additional trimming and filtering.

Subsequent processing followed the *Apogee* pipeline (**Supplementary Figure S3**), a modified version of the *Spaghetti* workflow optimized for full-length 16S rRNA gene nanopore reads (Latorre-Pérez et al., 2021). In brief, adapter trimming was performed with *Porechop* (Bonenfant et al., 2023), followed by length filtering using *NanoFilt 2.8.0* (De Coster et al., 2018) to retain reads between 1,200 and 1,800 bp. Read metrics were summarized with *NanoStat 1.6.0* (De Coster et al., 2018). Chimeric reads were identified via all-vs-all alignments using *Minimap2 2.28* (Li, 2018) with nanopore-specific settings (-x ava-ont, -f1000 to ensure a minimum mapped fragment length of 1000 base pairs for computational efficiency and accuracy, and a maximum seed distance of 500). The resulting PAF files were processed with *yacrd 1.0.0* (Marijon et al., 2020), applying a minimum coverage threshold of 0.4. High-quality reads were then aligned to the SILVA reference database (version 138) using *Minimap2 2.28* (Li, 2018). Alignments shorter than 500 bp were excluded using a custom Python script to ensure reliable taxonomic assignment. An amplicon sequence variant (ASV) table was constructed, listing read counts per taxon across samples. ASVs representing less than 1% of total reads were filtered using the *filterPAF* script in *Apogee*. A companion taxonomy table mapped ASV identifiers to taxonomic ranks from kingdom to species.

Functional profiling was performed using *PICRUSt2 2.6.2* (Douglas et al., 2020), which infers the metabolic potential of microbial communities from 16S rRNA gene data. Compared to PICRUSt1 (Langille et al., 2013), the updated PICRUSt2 pipeline incorporates representative sequences of *de novo* taxonomic clusters and improves prediction accuracy through Hidden State Prediction and expanded genome databases (Douglas et al., 2020). Predicted functions were annotated using KEGG orthologs and enzyme commission (EC) numbers (Kanehisa and Goto, 2000), enabling insight into putative microbial pathways for future experimental validation.

### 2.6 Statistical analysis

All statistical analyses were conducted in R (ver. 4.3.2). Alpha diversity indices were calculated using the *diversity* function from the *vegan 2.6.8* package (Oksanen et al., 2025), and true diversity matrices based on Simpson and Shannon indices were calculated as described by Jost (Jost, 2006). Data normality was assessed using the Shapiro-Wilk test. Due to non-normal distributions, generalized linear mixed models (GLMMs) were implemented via the *glmmTMB 1.1.10* package (Brooks et al., 2017) which leverages the Template Model Builder (TMB) framework. In the models, factors such as potato variety, field location, and sieving fraction were specified as fixed or random effects depending on the analytical objective. Bonferroni correction was applied to adjust *p*-values and control for Type I errors.

Beta diversity was assessed using the *vegan’s vegdist* function with the Robust Aitchison method to generate dissimilarity matrices, which were visualized via principal component analysis (PCA) using the *prcomp* function. Treatment effects on community composition were tested using PERMANOVA via the *adonis2* function, and pairwise comparisons were performed using *pairwise.adonis*.

To identify core microbiome members of different communities, we applied the abundance-occupancy framework described by Shade and Stopnisek (2019). In this approach, each treatment was considered a distinct ecological unit, and its corresponding replicates were treated as within-treatment samples. Occupancy for each ASV was defined as the proportion of replicates within a treatment in which the ASV was detected (range: 0-1), reflecting its consistency across replicates. Abundance was calculated as the mean relative abundance of each ASV within the treatment. ASVs with an occupancy of 1.0 (i.e., present in all replicates of a treatment) were considered consistently detected and prioritized for further analysis. ASVs were then ranked by both occupancy and abundance to identify those that were both prevalent and abundant, serving as potential core community members. The contribution of these core taxa to overall community structure was evaluated using Bray-Curtis similarity. To assess potential plant-beneficial functions, ASVs were screened against an in-house list of known PGPB compiled from published literature (Chao et al., 2016).

Differential abundance analysis of genera and species was performed using the ANCOM-BC2 package, which accounts for sample- and taxon-specific biases (Lin and Peddada, 2024). Significance was deemed at *p* = 0.05. Heatmaps were created using *pheatmap* (Kolde, 2025) to visualize the taxa differences across samples. Venn diagrams were created using *ggvenn* (Yan, 2025) to show the unique and shared ASVs across treatments.

Fast expectation-maximization microbial source tracking (FEAST) analysis was performed using the *FEAST 0.1.0* package (Shenhav et al., 2019) to estimate source contributions to sink samples based on an ASV count matrix data and metadata classifying sources and sinks. The output was a normalized matrix estimating proportional contributions. The analysis provided insight on what proportion of the 25 µm and 75 µm sieving fraction communities are represented by microbial sources associated with diseased tare soil versus unknown sources.

Network analysis was conducted using the Molecular Ecological Network Analysis (MENA) pipeline (http://ieg2.ou.edu/MENA) (Deng et al., 2012) to compare the microbial network between diseased wart and soil samples, and healthy soils. CLR-transformed ASV table and Pearson correlation were used to construct similarity matrices. Random Matrix Theory (RMT) thresholds were applied, and networks were visualized with *Gephi 0.10.1* (Bastian et al., 2009). Potential PGPB species identified as the network nodes were evaluated using heatmap visualization and *ANCOMBC2 2.6.0* (Lin and Peddada, 2024). Species that were significantly abundant in diseased communities were selected. Spearman correlation was conducted to assess the relationship between these species and *S. endobioticum* abundance quantified by qPCR.

Functional predictions from PICRUSt2 were analyzed using *DESeq2 1.44.0* (Love et al., 2014), and significantly enriched KEGG orthologs were passed to *clusterProfiler 4.12.6* (Yu et al., 2012) for gene set enrichment using the *gseKEGG function*. Human-related pathways were removed, and enriched functions were visualized using *cnetplot* in *clusterProfiler 4.12.6* (Yu et al., 2012) and *pathview 1.44.0* (Luo and Brouwer, 2013).

## 3 Results and discussion

### 3.1 Summary of sequencing reads

Nanopore sequencing generated approximately 24 million reads of the full length 16S rRNA gene region, yielding a total of 20 Gb of data across all samples. The average N50 was 1.6kb, and after error correction using the Apogee pipeline, high-quality reads had an average length of 1.5 kb. Median Phred quality scores reached 38, corresponding to a raw basecalling accuracy of 99.98%. In total, 3,995,215 reads and 69,333 amplicon sequence variants (ASVs) were recovered. Per sample, this corresponded to an average of 53,269 ± 22,391 reads and 4,837 ± 3,324 ASVs. Across all samples, 54 bacterial phyla, 140 classes, 356 orders, 636 families, 1,990 genera, and 4,651 species were identified (**Supplementary Table S4**). The sequencing files corresponding to the negative control (water) contained no detectable sequences, confirming that no contamination occurred during library preparation (**Supplementary Figure S4**).

### 3.2 Whole genome amplification preserved dominant taxa but introduced bias in rare taxon recovery

We first evaluated WGA-associated bias using a commercial mock bacterial community and eDNA from potato wart-infected soil collected in Newfoundland and Labrador. Canadian samples were used due to limited DNA availability from the Netherlands.

**Figure 1:**
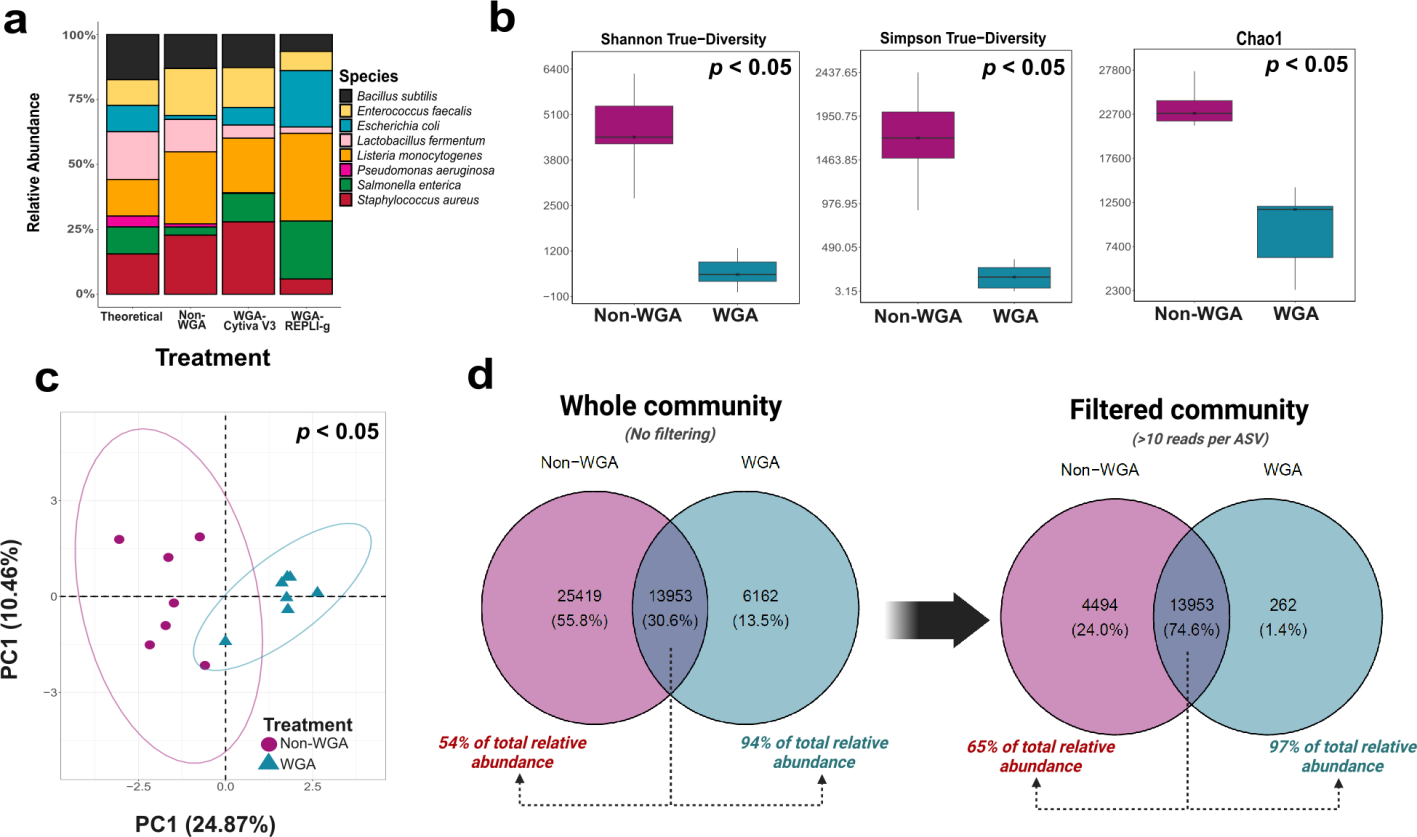
Effect of whole genome amplification on microbial recovery from mock and environmental samples. **(a)** Relative abundance profiles of eight bacterial species in the ZymoBIOMICS D6305 mock community under different treatments: Non-WGA, WGA using the Cytiva GenomiPhi kit, and WGA using the REPLI-g kit, compared against the theoretical composition. The Cytiva kit recovered all expected taxa, including low-abundance *Pseudomonas aeruginosa*, whereas REPLI-g failed to detect this species. **(b)** Alpha diversity metrics (Shannon true diversity, Simpson true diversity, and Chao1 richness index) for potato wart-infected soil samples, showing significantly reduced diversity in WGA-treated samples (*p* < 0.05). **(c)** Principal component analysis (PCA) of CLR-transformed ASV tables reveals distinct community composition between Non-WGA and WGA treatments (*p* < 0.05, PERMANOVA). **(d)** Venn diagrams comparing ASV overlap between Non-WGA and WGA samples before (left) and after (right) filtering out low-abundance ASVs (<10 reads). Without filtering, only 30.6% of ASVs were shared, accounting for 54% and 94% of total relative abundance in Non-WGA and WGA samples, respectively. After filtering, 74.6% of ASVs were shared, representing 65% and 97% of total relative abundance, indicating WGA preserved dominant taxa but distorted overall diversity and rare taxon representation.

All members of the mock community (ZymoBIOMICS D6305) were recovered from Cytiva-kit-amplified samples, including the least abundant species, *Pseudomonas aeruginosa* (theoretical abundance 0.4%), although it was detected at a reduced relative abundance (0.1%). In contrast, *P. aeruginosa* was not detected in samples amplified with the REPLI-g kit (**Figure 1a**, **Supplementary Table S5**). The relative abundances of other community members also deviated from the expected composition in both amplification treatments. Based on its more complete recovery of mock community taxa, the Cytiva kit was selected for all subsequent eDNA amplification in this study.

To further evaluate WGA-induced bias, paired non-WGA and Cytiva-WGA samples from potato wart-infected soils (n = 7 each) were compared. Non-WGA samples exhibited significantly higher alpha diversity (Simpson- and Shannon-based true diversity, Chao1 index; *p* < 0.05; **Figure 1b**), and PCA on CLR-transformed ASV tables revealed distinct community compositions between WGA and non-WGA treatments (*p* < 0.05, PERMANOVA; **Figure 1c**). By filtering low-abundance ASVs (<10 reads), 74.6% of ASVs were shared between WGA and non-WGA treatments, accounting for over 94% of total relative abundance in WGA samples, suggesting dominant taxa were reliably recovered (**Figure 1d**).

Although WGA preserved overall community structure, it introduced detectable bias affecting diversity estimates and low-abundance taxa detection. Unique ASVs in WGA samples contributed just 3-6% of total abundance but may reflect inflation of rare taxa or artifacts, despite low-abundance. Both Cytiva and REPLI-g kits employ multiple displacement amplification, which reduces bias compared to PCR-based methods but remains susceptible to representation errors due to stochastic primer binding and template accessibility, particularly in low-input samples (Raghunathan et al., 2005; Abulencia et al., 2006; Marine et al., 2014; Babayan et al., 2016; Gawad et al., 2016; Biezuner et al., 2021).

In addition to WGA-related distortions, potential biases introduced by Oxford Nanopore Technologies (ONT) sequencing must also be considered. ONT platforms are known to exhibit GC and/or AT content-dependent sequencing efficiency, variable ligation efficiency during library preparation, and basecalling errors, particularly in homopolymer regions (Chen et al., 2025). While recent advances in basecalling algorithms (e.g., Dorado super-accuracy models) have significantly improved read accuracy (Kuśmirek, 2023), error propagation in long reads can still affect taxonomic resolution and diversity estimates if not adequately corrected. These platform-specific limitations, combined with WGA biases, underscore the need for cautious interpretation of low-abundance taxa and alpha diversity metrics in WGA-treated ONT datasets. Where possible, validation using mock communities and read abundance filtering can mitigate some of these effects.

Overall, WGA enabled recovery of dominant bacterial taxa from low-input wart DNA, facilitating community profiling under quarantine constraints. However, it introduced compositional bias that affected rare taxa and alpha diversity estimates. These results emphasize the trade-off between data yield and taxonomic accuracy and the need to validate WGA-treated datasets when interpreting diversity or functional patterns.

### 3.3 Microbial partitioning between Spieckermann wart tissue fractions reveals physical structuring and taxonomic enrichment

To assess how microbes partition between host-associated and externally attached niches, we analyzed WGA-treated eDNA from Spieckermann bioassay-induced wart lesions to compare microbial communities across 25 µm and 75 µm fractions.

**Figure 2:**
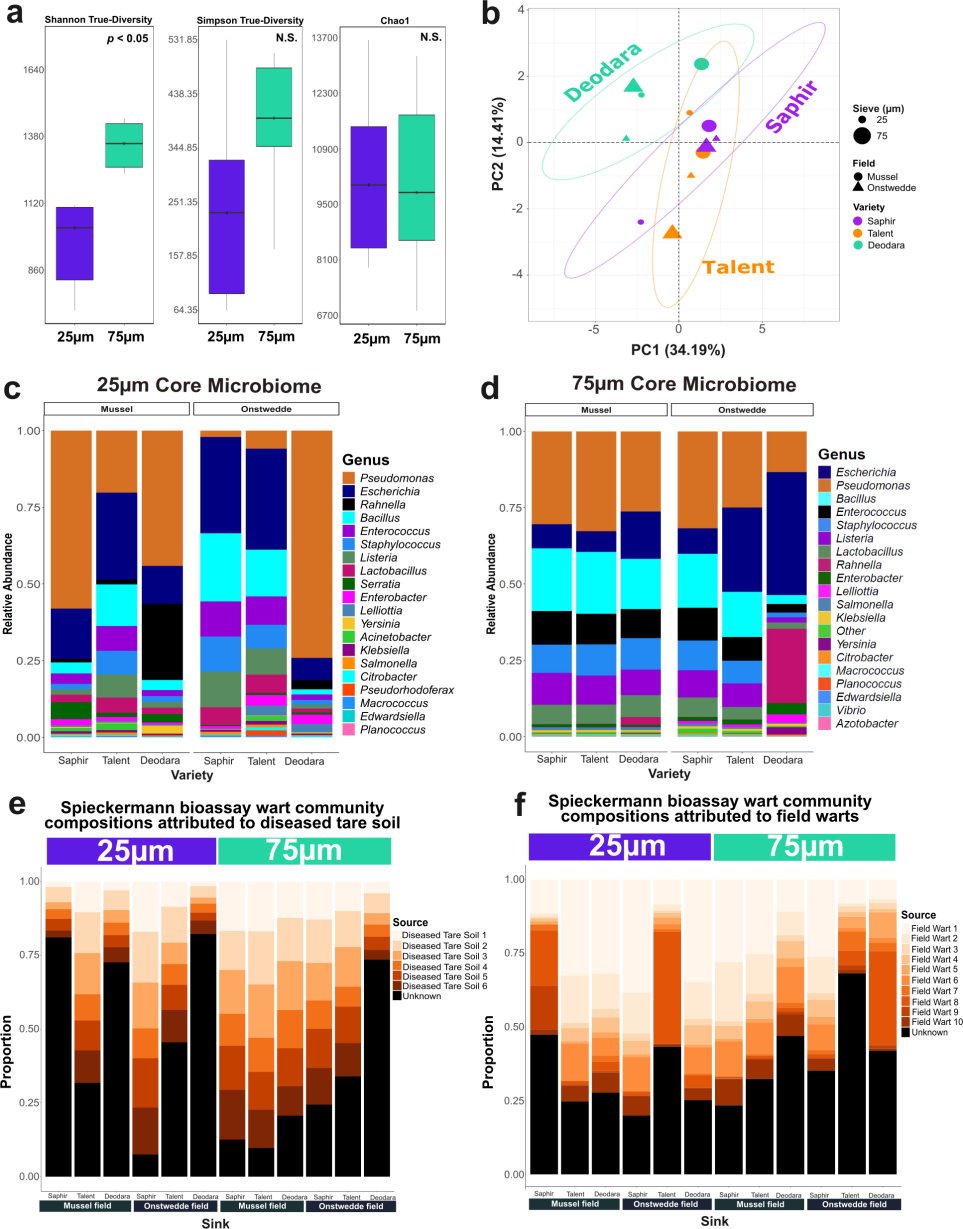
Microbial community differences between 25 and 75 µm sieving fractions of Spieckermann wart tissue. **(a)** Alpha diversity comparisons (Shannon-based true diversity, Simpson-based true diversity, and Chao1 richness index) reveal significantly higher Shannon diversity in the 75 µm fraction (*p* < 0.05), with no significant differences for Simpson or Chao1 metrics. **(b)** Principal component analysis (PCA) of CLR-transformed ASV data shows that sieve size did not significantly influence community structure (PERMANOVA *p* > 0.05), while cultivar had a significant effect (*p* = 0.012), with *Deodara* samples clustering separately from *Saphir* and *Talent*. **(c, d)** Relative abundance of the top 20 genera in the core microbiomes of the 25 µm **(c)** and 75 µm **(d)** fractions across samples, showing greater compositional consistency in the finer fraction and higher variability in the coarser fraction. **(e, f)** Microbial source tracking using FEAST estimates the proportion of each sample’s community originating from **(e)** diseased tare soil (source) and **(f)** field-derived warts (both in 75 µm fraction). The 75 µm fraction showed strong soil association, while the 25 µm fraction had a higher proportion of field wart sources, consistent with a more host-associated microbiome.

Samples were derived from two field sites in The Netherlands (Mussel and Onstwedde) and three susceptible cultivars (Deodara, Saphir, and Talent), with both fractions processed in parallel. The 75 µm fraction showed significantly higher Shannon-based true diversity (*p* < 0.05; **Figure 2a**), likely reflecting the inclusion of rhizoplane- or soil-associated taxa. In contrast, the finer 25 µm fraction harbored fewer taxa but displayed higher inter-sample compositional heterogeneity. No significant differences in Simpson diversity or Chao1 richness were observed, suggesting that variation stemmed primarily from rare taxa rather than shifts in dominant members.

PCA and PERMANOVA revealed no significant compositional differences between sieve sizes or field sites (*p* > 0.05; **Figure 2b**). However, microbial composition differed significantly by cultivar (PERMANOVA *p* = 0.012), with Deodara samples forming a distinct cluster (pairwise PERMANOVA *p* < 0.05). This highlights the influence of host genotype such as cultivar-specific susceptibility to *S. endobioticum* pathotypes (van de Vossenberg et al., 2022b) and pedigree differences (van Berloo et al., 2007) in shaping wart-associated microbiota, even under standardized conditions. In this context, variation in microbial composition may also contribute to differences in the success rate of the Spieckermann bioassay, which has been used for over a century, yet this microbiome aspect has been largely overlooked (van de Vossenberg et al., 2019a). These findings reinforce our overarching hypothesis that *S. endobioticum* reshapes microbial communities via both pathogen stress and host genetic filtering, in line with broader trends in potato-microbiome-pathogen interactions (Qing et al., 2024; Yang et al., 2024).

Core microbiome analysis identified 237 and 294 ASVs in the 25 µm and 75 µm fractions, respectively, accounting for ~15-16% of total abundance. The 75 µm fraction exhibited greater compositional consistency across cultivars and sites, suggesting environmental buffering and dominance of generalized taxa. In contrast, the 25 µm fraction was more variable, potentially enriched in microbes tightly associated with resting spores or wart internal tissues. Despite these differences, both fractions shared 12 genera of potential biocontrol interest, including *Pseudomonas, Bacillus, Klebsiella, Enterobacter, Citrobacter*, and *Staphylococcus* (**Figures 2c, d**). While not differentially abundant between fractions (ANCOM-BC2, *p* ≥ 0.05), these genera include known plant-growth-promoting bacteria (PGPB) and biocontrol agents, though care must be taken in inferring function from genus-level resolution.

FEAST-based microbial source tracking revealed that the 75 µm fraction closely resembled diseased tare soils, with >70% of community origins attributed to soil in five of six samples. In contrast, three of six 25 µm samples had >75% of their predicted origin classified as “unknown,” indicating limited soil contribution and stronger host association. Notably, both fractions showed substantial attribution to field wart microbiomes, with the 25 µm fraction slightly more enriched (50-75%) than the 75 µm fraction (30-75%) (**Figures 2e, f**). These results suggest that finer fractions capture more host-filtered or internalized communities, while coarser fractions retain more transient, environmental taxa.

In summary, the 25 µm and 75 µm fractions represent distinct yet overlapping niches: the finer fraction captures host-associated and endophytic taxa relevant to wart-specific interactions, while the coarser fraction reflects more diverse, soil-derived reservoirs of potential PGPB and suppressive microbes.

### 3.4 Bioassay-derived wart microbiomes recapitulate diversity but differ in composition from field samples

To test whether Spieckermann bioassays replicate field wart microbiomes, we compared alpha and beta diversity between bioassay- and field-derived 75 µm samples to evaluate the bioassay’s suitability as a controlled model system for microbiome study.

**Figure 3:**
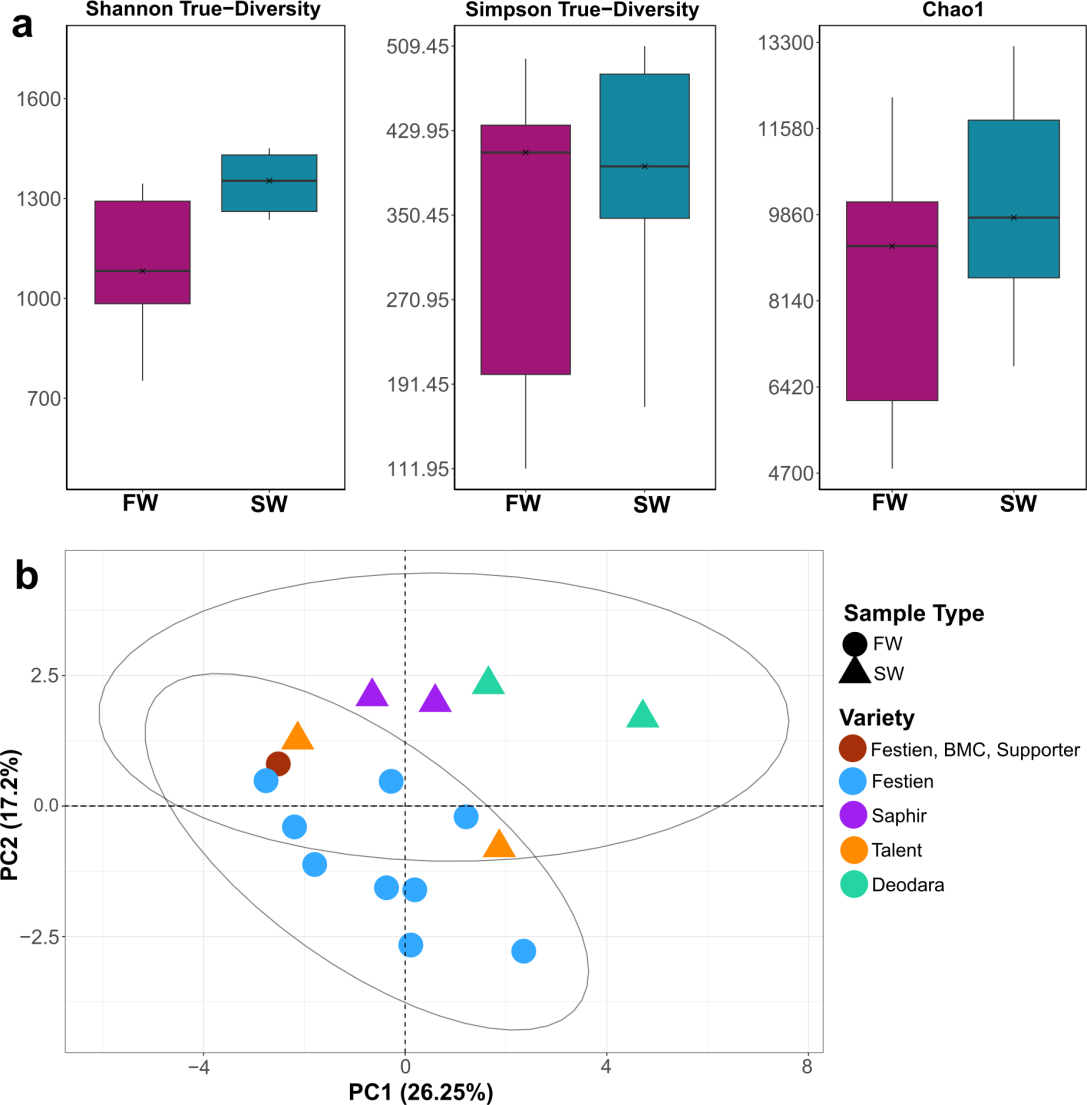
Alpha and beta diversity of field- and bioassay-derived wart microbiomes in 75 µm fractions. **(a)** Alpha diversity indices (Shannon-based true diversity, Simpson-based true diversity, and Chao1 richness) show no significant differences between field (FW) and Spieckermann bioassay-derived (SW) wart samples (*p* > 0.05), indicating similar levels of richness and evenness across systems. **(b)** Principal component analysis (PCA) of CLR-transformed ASV data reveals significant differences in community composition between FW and SW samples (*p* < 0.05, PERMANOVA). FW, field-derived; SW, bioassay-derived.

Alpha diversity metrics (Shannon, Simpson, and Chao1) showed no significant differences between field and bioassay samples (*p* > 0.05; **Figure 3a**), indicating that overall microbial richness and evenness were comparable across systems. However, PCA revealed significant differences in microbial community composition (*p* < 0.05; PERMANOVA; **Figure 3b**). We observed that host genotype had a significant impact on bioassay-derived wart microbiome (**Figure 3b**); the absence of overlapping host genotypes between field and lab conditions, therefore, limits our ability to resolve whether the observed differences are driven by environmental context (field vs. lab), host genotype, or their interaction. Nevertheless, **Figures 2e, f** showed that the 75 µm fraction of bioassay-derived wart microbiomes showed strong similarity to diseased tare soils and also retained substantial resemblance to field-grown warts, with 30-75% of its composition attributed the microbiomes of field samples. This dual attribution supports the notion that the 75 µm fraction includes both loosely associated soil taxa and residual host-associated communities.

In summary, the bioassay reflects wart-associated diversity but differs compositionally from field samples, likely due to host genotype and environment. Despite these differences, it remains a useful controlled model, and genotype-matched studies are needed to better separate host and environmental effects. The consistent variety effect across systems reinforces host genotype as a key microbiome driver.

### 3.5 Compartment-aware prioritization of biocontrol candidates: contrasting microbiomes in wart tissues and diseased soils

To assess their potential as biocontrol reservoirs against S. endobioticum, we compared microbiomes of field wart tissues and diseased tare soils (both 75 µm fractions). Wart fractions were enriched with pathogen spores, whereas infected soils contained only trace amounts.

Alpha diversity indices differed insignificantly between wart and tare soil samples (*p* > 0.05); however, wart samples exhibited greater variability, likely reflecting their heterogeneous origins ranging from fresh to decaying warts collected across different field sites (**Supplementary Figure S5a**). Beta diversity analysis revealed significant differences in community composition (*p* < 0.05, PERMANOVA; **Supplementary Figure S5b**), supporting the existence of compartment-specific microbial assemblages.

**Figure 4:**
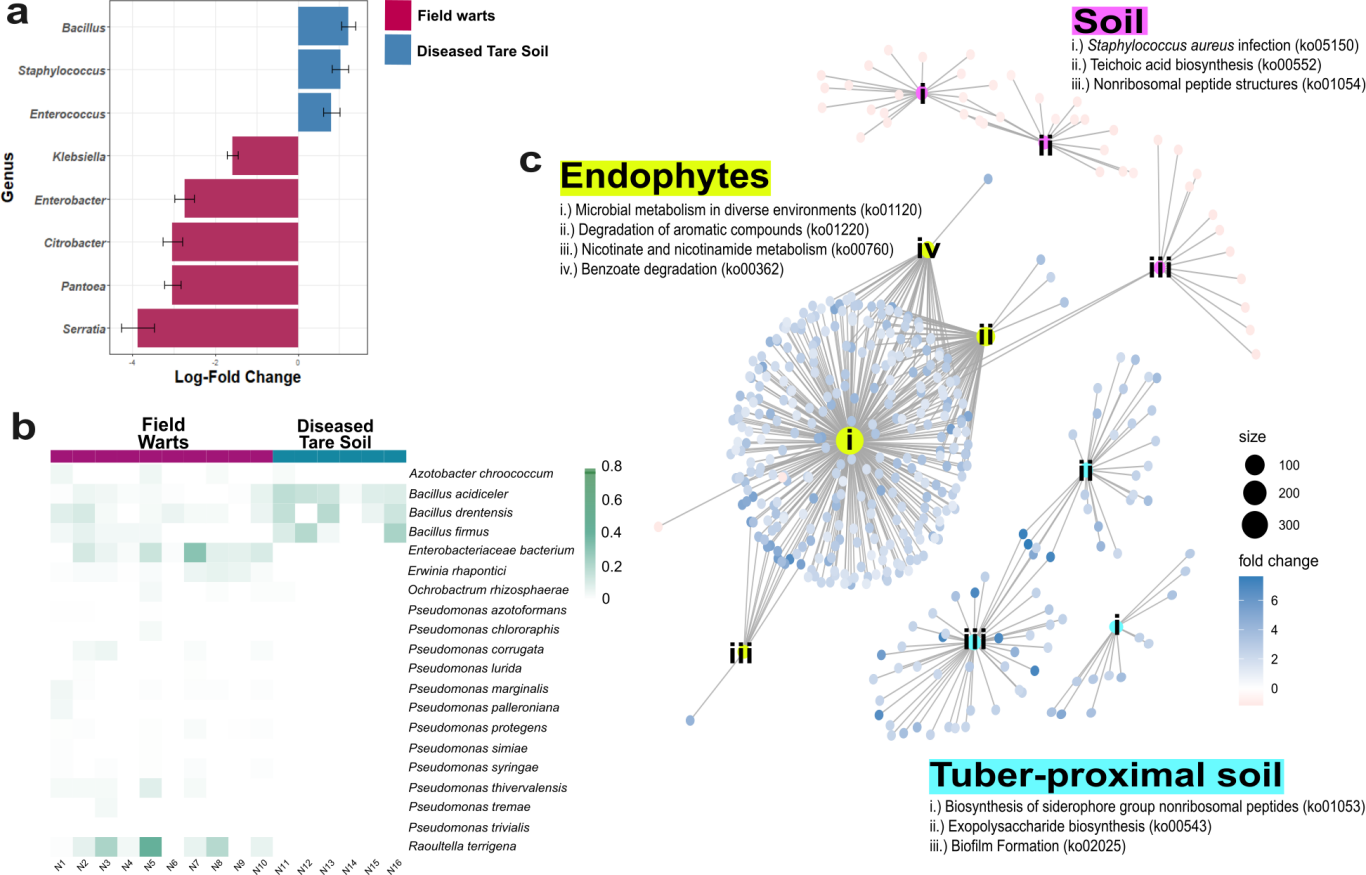
Taxonomic and functional differences between bacterial communities in field wart tissues and diseased tare soils. **(a)** Differential abundance of bacterial genera between wart and diseased tare soil samples identified using ANCOM-BC2. Bars indicate log-fold change in centered log-ratio (CLR) transformed abundance, with positive values enriched in wart tissues and negative values enriched in tare soils. **(b)** Heatmap of CLR-transformed relative abundances of differentially abundant species from both compartments that possess annotated chitin-degradation pathways, including chitinases, chitin deacetylases, and lytic monooxygenases (**Supplementary Table S6**). **(c)** Gene set enrichment analysis (GSEA) of KEGG orthologs shows distinct functional enrichments across compartments. Circle size represents -log10(*p*) values, and direction of enrichment is based on log-fold change: positive values (in blue) indicate enrichment in field wart tissues, while negative values (in red) indicate enrichment in diseased tare soils. Selected pathways are grouped by ecological relevance (e.g., soil-, tuber-proximal, or endophyte-associated functions) based on prior literature.

Wart tissues hosted more variable and host-associated taxa, including *Klebsiella*, *Enterobacter*, *Citrobacter*, *Pantoea*, and *Serratia*, many with known endophytic traits or stress-associated functions such as siderophore production (ko01053), biosynthesis of exopolysaccharides (ko00543), biofilm formation (ko02025), and aromatic compound degradation (ko01220) (Purkayastha et al., 2018; Hennessy et al., 2020; Kharat et al., 2022; Akber et al., 2023; **Figure 4c**). *Klebsiella oxytoca*, for example, is known to induce systemic resistance via antioxidant signaling (Elsharkawy et al., 2022a), while *Enterobacter* spp. had previously recovered in high abundance from potato scabby tubers (Macharoen et al., 2024). These traits are consistent with enhanced microbial persistence, detoxification, and host colonization capacity under stress conditions (Maindad et al., 2014; Khalil et al., 2019).

In contrast, diseased tare soils exhibited more consistent microbial profiles (**Supplementary Figure S6**) enriched in *Bacillus*, such as *B. acidiceler, B. drentensis, and B. firmus* (ANCOM-BC2 analysis, *p* < 0.05, **Figures 4a, b**), with known functions in chitin degradation, antimicrobial compound synthesis, and plant growth promotion (Ding et al., 2005; Mahmood et al., 2016; Yousuf et al., 2017; Saxena et al., 2020; Alshammari et al., 2024). For example, the enrichment of nonribosomal peptide biosynthesis pathway (ko01054) (**Figure 4c**), a hallmark of bioactive compound production, has been found associated with suppressive soils (Roongsawang et al., 2011; Wang et al., 2018; Rabbee et al., 2019; Tracanna et al., 2021). These taxa and functionalities are broadly active against soilborne pathogens and are easier to culture (Zhao et al., 2018; Saxena et al., 2020; Tracanna et al., 2021), but their suppressive potential may be more generalized (Brown et al., 2013; Chen et al., 2013; Alijani et al., 2019; Buchholz et al., 2019; Gao et al., 2020) rather than specifically effective against *S. endobioticum*.

Notably, PICRUSt2 predicted the presence of chitin degradation pathways in both compartments, highlighting the potential for isolating bacterial taxa capable of targeting the chitin-rich cell walls of *S. endobioticum* (Hayes et al., 2017; van de Vossenberg et al., 2019b). Warts were enriched in chitin deacetylase (ko01452), putative chitinase (ko03791), and bifunctional chitinase/lysozyme (ko13381), while tare soils were enriched in lytic chitin monooxygenase (ko21713) (**Supplementary Table S6**), suggesting divergent enzymatic strategies for chitin breakdown.

In summary, wart tissues contained variable, host-associated taxa with stress-adaptive traits, while diseased soils hosted consistent, culturable taxa with broad antagonistic potential. Both showed potential chitinolytic activity, indicating complementary niches for targeting *S. endobioticum*.

### 3.6 Soil microbiome legacy and PGPB reservoirs in wart-infested and descheduled fields with implications for long-term wart suppression

We examined diseased and descheduled soils to determine whether long-term microbial legacy and recovery reveal traits linked to wart suppression (fifth research question). Descheduled soils, once infested with *S. endobioticum* but rendered disease-free after prolonged non-host rotations (EPPO Bulletin, 2017a), provide a unique lens on legacy effects without active infection. In potato wart-infested soils, although spores persist at low levels, their residual pathogenicity implies ongoing host-microbe interactions. Drawing on concepts of keystone pathogen effects (Hajishengallis et al., 2012), microbiome restructuring, and ecological memory, we hypothesized that even minimal pathogen pressure can drive lasting shifts in community structure and function. Our analysis focused on bacterial guilds with potential *S. endobioticum*-specific or broad-spectrum antagonism.

**Figure 5:**
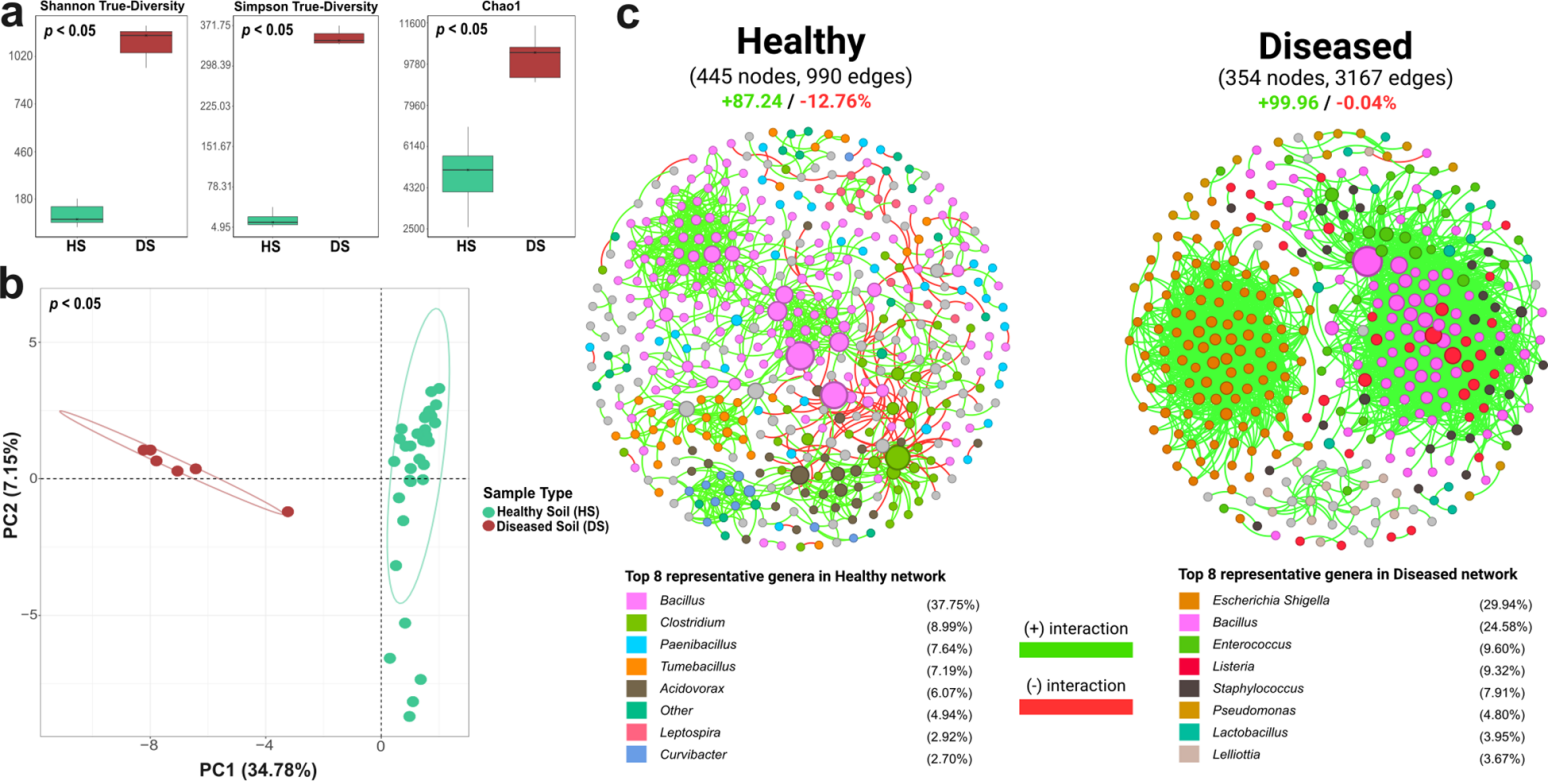
Microbial diversity and network structure differ markedly between healthy (HS) and diseased (DS) soil communities. **(a)** Alpha diversity indices show significantly higher richness and evenness in DS compared to HS samples (*p* < 0.05), potentially reflecting microbial proliferation under pathogen pressure. **(b)** Beta diversity based on PCA of CLR-transformed ASV profiles reveals distinct clustering between HS and DS communities (*p* < 0.05, PERMANOVA), indicating compositional divergence. **(c)** Co-occurrence networks highlight structural contrasts: the DS network forms dense, highly clustered modules with short path lengths, but shows lower connectedness and modularity, suggesting locally reinforced but ecologically narrow interactions. In contrast, the HS network displays higher modularity, greater connectedness, and more dispersed interactions, reflecting a resilient and functionally compartmentalized microbial ecosystem.

Corroborating our findings on compartment-specific microbial assemblages from the 25 µm and 75 µm wart fractions, diseased soils processed using the coarser 75 µm sieve exhibited significantly higher alpha diversity than descheduled (healthy) soils processed using the finer 25 µm sieve (**Figure 5a**). This result must be interpreted with caution due to the non-equivalent sieving fractions. The 75 µm fraction likely retains more root debris and spore-laden particles which can harbor diverse, pathogen-associated microbiota (Hemkemeyer et al., 2018; Dittrich et al., 2024). Most studies have reported higher microbial diversity in healthy soils versus when samples were processed in parallel (Jia et al., 2022; Yang et al., 2024). Descheduled soils, however, displayed higher compositional heterogeneity across samples (**Figure 5b**), with lower representation in wart-associated microbiomes based on FEAST source-tracking (**Supplementary Figure S7**). Such observations are, at least partially, attributed to the impacts of various long-term non-host rotation regimes and other field operational practices, which have not yet been disclosed to us due to confidentiality under quarantine regulations. This also highlights another implication of host influence on soil microbiomes (Mészárošová et al., 2024).

Co-occurrence network analysis revealed stark contrasts in microbial community structure between diseased and descheduled soils (**Supplementary Table S7**), albeit with caution due to differing sieving fractions (75 µm vs. 25 µm) and field operations. The healthy soil network exhibited greater modularity (0.777), connectedness (0.648), geodesic efficiency (0.988), along with higher betweenness centralization (CB = 0.242), and higher proportion of competitive (negative) correlations which are features of mature, functionally partitioned ecosystems supporting regulatory stability (Coyte et al., 2015). In contrast, the diseased soil network showed dense clustering (avgCC = 0.462), shorter path lengths (GD = 2.459), and near-complete dominance of positive correlations (99.96%), yet had lower connectedness (0.372) and modularity (0.449). These features suggest insular microbial modules with weak regulatory feedback and limited cross-module integration which are conditions that may foster a disrupted, less resilient soil ecosystem. Such contrasting ecological network representations of course is largely influenced by soil physicochemical properties and organic matter, local climate and agronomic practices, and timing of sampling of crop lifecycle, etc (Banerjee et al., 2018; Mandakovic et al., 2018; Pechlivanis et al., 2024; Wei et al., 2024), besides different levels of disease pressures.

**Figure 6:**
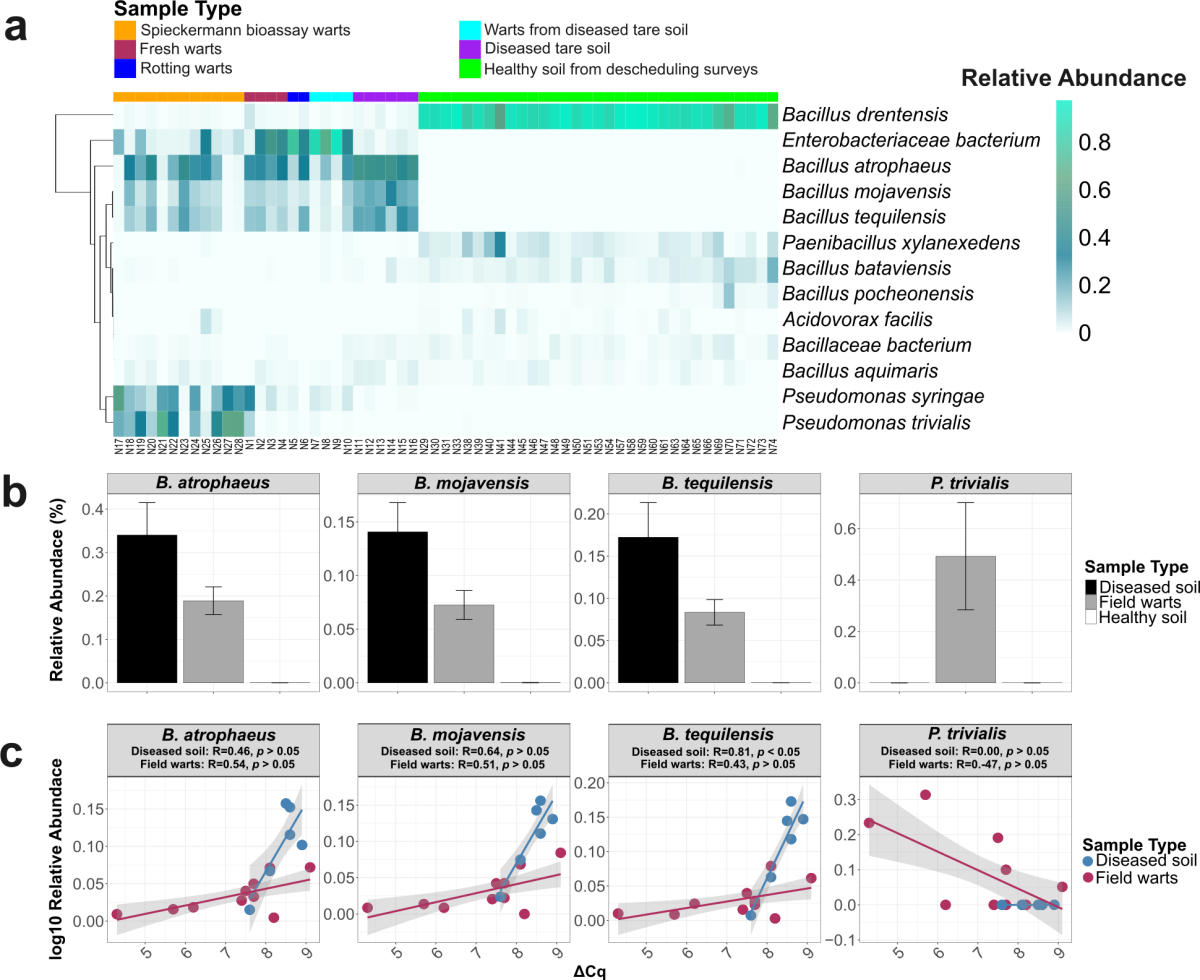
Species-level microbial patterns in diseased versus healthy soils, highlighting potential biocontrol taxa. **(a)** Heatmap of centered log-ratio (CLR) transformed relative abundances of key bacterial species identified from co-occurrence network analysis, stratified by sample type. **(b)** Relative abundance profiles of significantly enriched *Bacillus* and *Pseudomonas* plant growth-promoting bacteria (PGPB) in diseased versus healthy soils. **(c)** Correlation analysis between the abundance of these potential PGPB species and *S. endobioticum* levels (represented as ΔCq values from qPCR). Trend lines indicate species-specific association patterns, with several *Bacillus* spp. showing positive correlations and *Pseudomonas trivialis* exhibiting a negative relationship.

From a BCA reservoir perspective, *Bacillus* was the only genus consistently present in both networks, comprising 24.6-37.8% of all nodes (**Figure 5c**). Several species, such as *B. atrophaeus*, *B. mojavensis*, and *B. tequilensis*, were enriched in diseased soils and wart tissues (**Figure 6a, b**) and are known for antifungal activity, ISR induction (Cao et al., 2025), enhanced resource use efficiency (Sdiri Ghidawi et al., 2025), biofilm formation, pathogen suppression (Liu et al., 2020; Xu et al., 2021; Bi et al., 2025), and had shown ability to reduce disease severity by up to 60% across diverse cropping systems (Miljaković et al., 2020; Saxena et al., 2020; Mahapatra et al., 2022; Serrão et al., 2024). Moreover, some *Bacillus* spp. showed positive correlations with *S. endobioticum* abundance in field wart samples and diseased tare soils (**Figure 6c**). The presence of *Bacillus* spp. in both diseased and descheduled soils reinforces its status as a ubiquitous, beneficial soil taxon (Saxena et al., 2020), suggesting that biocontrol activity may already be occurring *in situ* and could represent a microbial legacy shaped by prior disease pressure.

By contrast, *Pseudomonas trivialis* was specifically enriched in wart tissues (**Figure 6b**), suggesting it may be responding to localized stress or plant-derived signals within infected tissue. Despite its known plant growth-promoting and biocontrol properties (Abd El-Aziz and Bashandy, 2019), *P. trivialis* exhibited a negative correlation with *S. endobioticum* abundance (**Figure 6c**). This may be due to the inability of *P. trivialis* to form endospores unlike *Bacillus* spp (Beskrovnaya et al., 2021), which may limit its persistence under fluctuating or disease-altered soil conditions. These observations potentially reflect reduced compatibility of *P. trivialis* with pathogen-dominated microenvironments. However, previous studies suggest that *Pseudomonas* spp. can be recruited or stabilized by *Bacillus*-mediated interactions (Sun et al., 2022; Zhang et al., 2023), raising the possibility that co-inoculation strategies may enhance its role in biocontrol or resilience under wart pressure.

**Figure 7:**
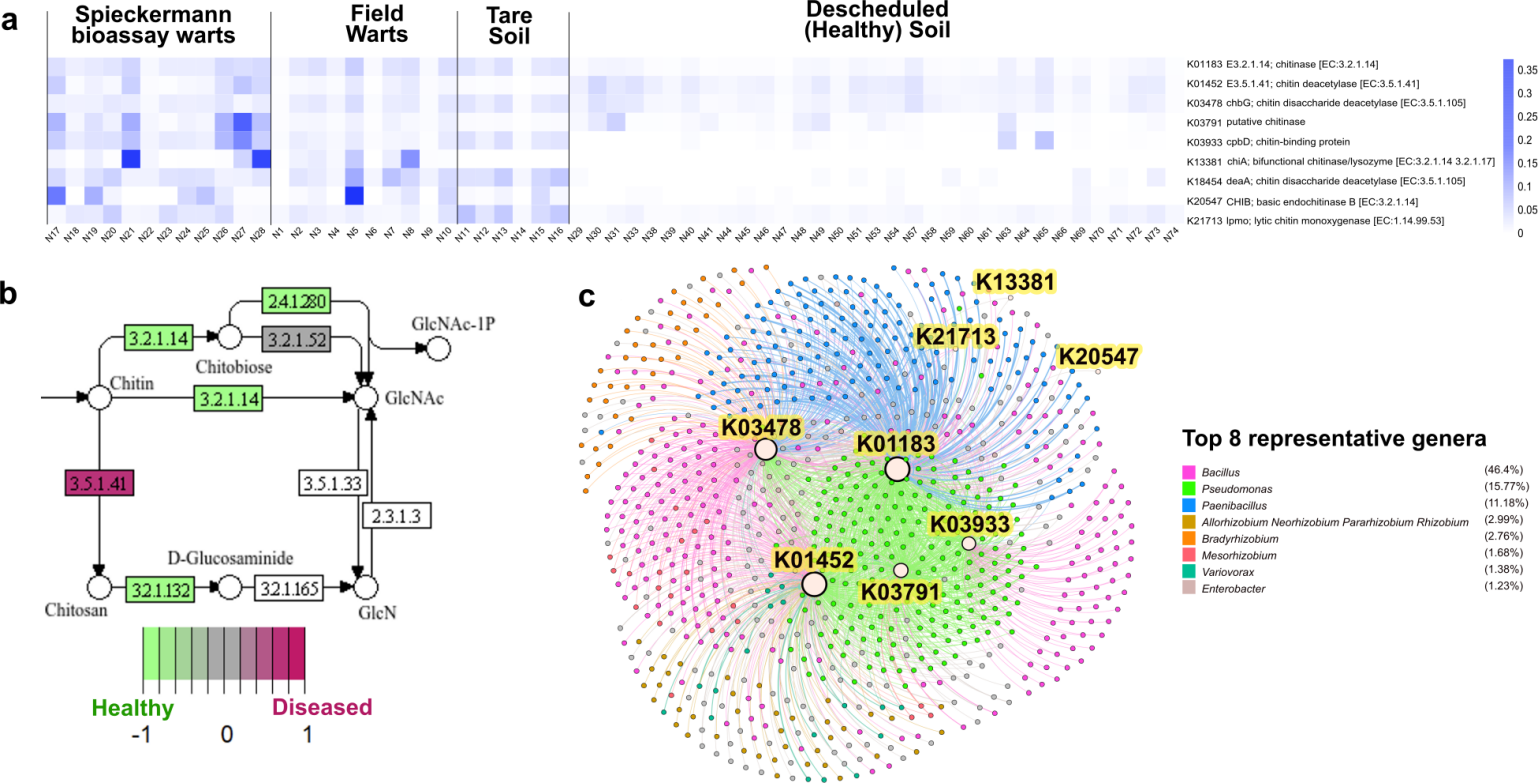
Functional prediction of chitin metabolic genes and pathways associated with the diseased and healthy systems. **(a)** Heatmap displaying differentially abundant genes involved in chitin metabolism across all sample types (*p* < 0.05). **(b)** The KEGG orthologues and corresponding protein with enzyme commission (EC) numbers are shown associated with chitin metabolism in the amino sugar and nucleotide sugar metabolism pathway (ko00520). Red and green background of each enzyme represents increased and decreased copy numbers in diseased systems in relation to descheduled soils, respectively. **(c)** Association network showing the links between KEGG orthologues involved in chitin metabolism and contributing bacterial ASVs coloured by genus.

Functional prediction showed that nine genes involved in chitin metabolism (*padj* < 0.05) were found in both diseased and healthy communities, with some contributing taxa being *B. drentensis, B. mojavensis, B. tequilensis* (**Supplementary Tables S8** and **Supplementary Figure S8**), corroborating with taxonomic recovery. While studies on *B. drentensis* remains limited, both *B. mojavensis* and *B. tequilinesis* have been reported for their ability to produce chitinase or chitosanase in addition to other bioactive compounds (Mounia et al., 2014; Liaqat et al., 2018; Baard et al., 2023; Afzal et al., 2025). The enriched chitin metabolism pathways in disease systems were likely driven by host and soil properties in additional to their response to *S. endobioticum*, particularly in wart tissues (Garbeva et al., 2011; Supronienė et al., 2023). Pathview analysis revealed that the chitin deacetylase-coding gene K01452 (EC 3.5.1.41) was more abundant in diseased systems than in the descheduled soils (**Figures 7a, b**). This enzyme hydrolyzes N-acetamido groups in N-acetyl-D-glucosamine residues of chitin, a key step in chitin degradation (Araki and Ito, 1974). The network (**Figure 7c**) demonstrated the association between the KEGG orthologues involved in chitin metabolism and bacterial ASVs, which predominantly belonged to genera *Bacillus*, *Pseudomonas*, and *Paenibacillus*. Strains of these genera have been studied for their ability to produce chitinase and act as biocontrol agents against phytopathogens (Govindasamy et al., 2010; Elbouazaoui et al., 2022; Dobrzyński and Naziębło, 2024).

Together, our results show contrasting taxonomic and functional profiles between diseased and descheduled (healthy) systems. While some differences may reflect sieve size, field practices, or crop histories, diseased compartments (warts and infested soils) were enriched for xenobiotic-degradation pathways and stress-adapted taxa, consistent with microbial responses to pathogen pressure. Descheduled soils exhibited more stabilized network structure and traits linked to generalized suppressiveness. Notably, functions inferred via PICRUSt2 are hypothesis-generating rather than confirmatory; they prioritize targets for isolation and testing. These complementary signatures point to opportunities to isolate BCAs that are either potato-wart-targeted or broadly antagonistic.

## 4 Conclusion and future perspectives

This study presents the first microbiome characterization of the potato wart disease pathosystem (*Synchytrium endobioticum*-*Solanum tuberosum*), a strictly regulated and previously unexplored quarantine system with limited global research access. By integrating wart tissues, tare soils, and descheduled soils, we identified microbiome patterns and potential microbial reservoirs across a gradient of disease pressure, providing essential starting points for hypothesis-driven research into disease suppression and biocontrol. The integration of Whole Genome Amplification (WGA), long-read sequencing, and co-occurrence network and functional prediction analyses establishes an ecological and methodological framework that can guide similar studies under quarantine or biosafety constraints. Collectively, this work advances both the fundamental understanding and applied research capacity for a previously inaccessible pathosystem.

Our central hypothesis, that *S. endobioticum* infection reshapes the potato-associated microbiome, potentially favoring taxa with biocontrol potential, was partially supported. Consistent enrichment of putative PGPBs such as *Bacillus* and *Pseudomonas* spp. suggests possible suppressive roles, though their ecological functions remain unresolved. Network fragmentation in diseased soils and higher modularity in descheduled soils indicate pathogen-driven restructuring, yet causality cannot be inferred. The use of WGA, while necessary for low-input samples, introduced recognized biases that we explicitly acknowledge. Functional inferences drawn from predictive metagenomics such as the use of PICRUSt2 or FAPROTAX (Louca et al., 2016; Douglas et al., 2020) and network topology (Lupatini et al., 2014; Cardona et al., 2016; MatChado et al., 2021) offer valuable leads but require experimental validation, especially given the methodological caveats (e.g., non-uniform sieving fractions and variable field histories) that limit taxonomic comparability. Given the logistical constraints of working with a quarantine pathogen, predictive approaches remain indispensable for hypothesis generation. Our findings identify candidate taxa for targeted isolation and functional testing, forming a foundation for subsequent *in vitro*, *in planta*, and field-level biocontrol validation. As such, we view this study as a necessary first step in a broader biovigilance and biocontrol discovery pipeline for potato wart disease, which lays a foundational framework for developing microbiome-informed strategies to manage potato wart disease.

**Figure 8:**
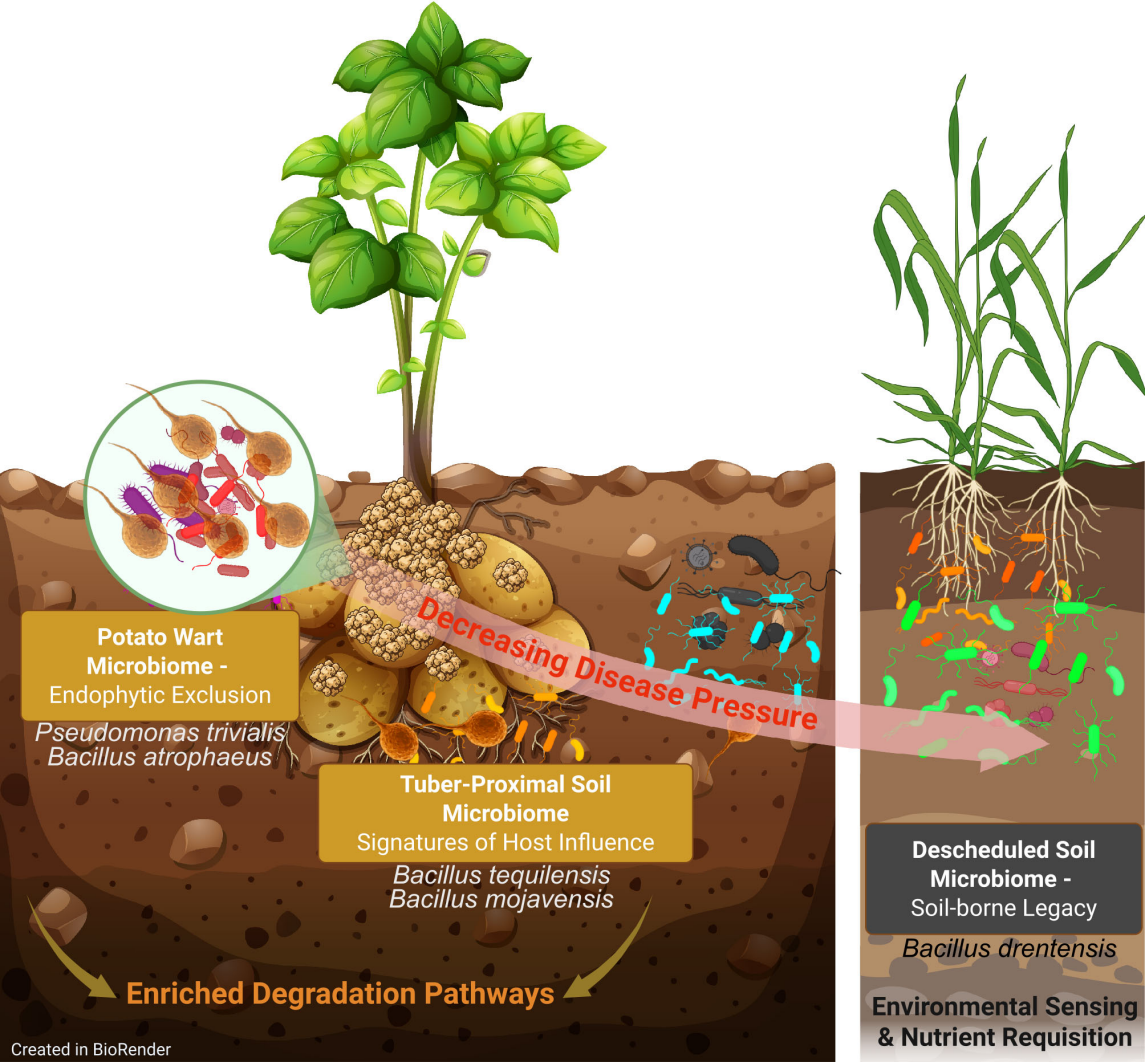
Conceptual model of potato wart gradient and associated microbiomes as biocontrol agent reservoirs. The potato wart compartment (left) represents high disease pressure, enabling intimate interaction between *Synchytrium endobioticum* and its associated microbiota. This niche is enriched in specialized taxa such as *Pseudomonas trivialis* and *Bacillus atrophaeus*, which may contribute to pathogen-specific suppression via mechanisms like endophytic exclusion. The tuber-proximal soils harbor transitional microbial structuring and functional signatures, enriched in plant-interactive taxa such as *Bacillus tequilensis* and *B. mojavensis* that may offer both generalist and targeted BCA activity, indicative of microbial recruitment influenced by the host-pathogen interface. These diseased systems are also enriched in xenobiotic and aromatic compound degradation pathways. At the opposite end of the gradient, descheduled soils (right), are typically managed under long-term non-host crop rotations (10–20 years) and harbor a legacy microbiome dominated by broad-spectrum BCAs such as *Bacillus drentensis*. These wart-free systems support soil-borne resilience and general disease suppression through enhanced environmental sensing, nutrient requisition, and microbial competition. Together, this gradient highlights the functional diversity and ecological plasticity of BCA reservoirs shaped by potato wart disease history and field management practices.

We propose a conceptual model illustrating a gradient of disease pressure and microbiome composition as reservoirs of biocontrol agents (BCAs) (**Figure 8**). In high-pressure wart compartments, taxa such as *Pseudomonas trivialis* and *Bacillus atrophaeus* may contribute to pathogen-specific suppression via endophytic exclusion. Transitional niches, including geocaulosphere (pre-harvest) and tare (post-harvest) soils, harbor plant-interactive taxa (e.g., *Bacillus tequilensis* and *B. mojavensis*) linked to host-mediated microbial recruitment (Roquigny et al., 2018; Shi et al., 2025). These systems are also enriched in xenobiotic and aromatic compound degradation pathways. In contrast, long-term descheduled soils, typically under non-host rotations, harbor broad-spectrum BCAs such as *B. drentensis*, contributing to general soil suppressiveness through enhanced sensing, nutrient competition, and resilience. Together, these gradients illustrate the ecological plasticity of BCA reservoirs shaped disease history and management practices.

While this study focused on bacterial communities, a complete understanding of potato wart suppression requires integrating fungi, oomycetes, archaea, and protists. Cross-kingdom interactions influence both disease progression and suppression, for instance, antagonism between *Pseudomonas fluorescens* and *Rhizoctonia solani* via iron competition (Elsharkawy et al., 2022b), or synergistic bacterial-fungal effects on plant immunity and nutrient uptake (Ansari et al., 2024). Fungal endophytes such as *Cladosporium* and *Trichoderma* spp. have also been associated with variable pathogen resistance in potatoes (Niyokwizigirwa, 2022), Future multi-kingdom metabarcoding or shotgun metagenomics will be essential to disentangle these cross-domain interactions and develop a holistic view of the phytomicrobiome influencing pathogen persistence and host resilience.

We are continuing to investigate microbiome dynamics under disease-conducive and suppressive conditions, as well as under targeted interventions that modify soil microbiota. These studies will employ statistically robust experimental designs and standardized sampling across field types, coupled with mechanistic assays and *in vitro*/*in planta* validation of candidate PGPB. We will continue addressing technical caveats such as WGA bias to preserve original microbial signals. Only through this integrative, hypothesis-driven approach can we transition from ecological inference to actionable biocontrol strategies for sustainable potato wart management.

## Data Availability

Raw sequencing data of the full length 16S rRNA gene are available under BioProject PRJNA1300411, Biosample accession SAMN50379165 - SAMN50379243.
