# Supplementary material for: Decoding microbiome responses to quarantine potato wart disease: first insights into suppression and biocontrol by full-length 16S rRNA gene profiling and functional prediction
Source: Front Plant Sci. 2026 Feb 3;16:1707759. doi: 10.3389/fpls.2025.1707759 (PMC12909553; doi:10.3389/fpls.2025.1707759)
Supplement: Supplementary file 1 [file DataSheet1.pdf]

frontiers

Supplementary Figures

Decoding Microbiome Responses to Quarantine Potato Wart Disease: First Insights into Suppression and Biocontrol by Full-Length 16S rRNA Gene Profiling and Functional Prediction

Running title: Microbiome Response to Quarantine Potato Wart

Ishraq Akbar [1,7], Yichao Shi [1], Bart T.L.H. van de Vossenberg [2], Theo A.J. van der Lee [3], Lang Yao [4], Xiang Li [5], Jiacheng Chuan [5], Linda E. Jewell [6], Hai D.T. Nguyen [1], Wen Chen [1,7*]

[1]Ottawa Research & Development Centre, Agriculture & Agri-Food Canada (AAFC), Ottawa, ON K1A 0C6, Canada
[2]Netherlands Institute for Vectors, Invasive Plants and Plant Health, National Plant Protection Organization, Netherlands Food and Product Safety Authority, Geertjesweg 15 6706EA Wageningen, Netherlands
[3]Biointeractions and Plant Health, Wageningen University & Research, Droevendaalsesteeg 4, 6708PB, Wageningen, the Netherlands
[4]The Ottawa Laboratory (Carling), Canadian Food & Inspection Agency (CFIA), Ottawa, ON K1A 0C6, Canada
[5]The Charlottetown Laboratory, Canadian Food & Inspection Agency (CFIA), Charlottetown, PE C1A 5T1, Canada
[6]St. John's Research and Development Centre, Agriculture & Agri-Food Canada, St. John's, NL A1E 6J5, Canada
[7]Department of Biology, University of Ottawa, Ottawa, ON K1N 9A7, Canada

*** Correspondence:**
Wen Chen, wen.chen@agr.gc.ca

Keywords: Potato wart, microbiome, nanopore sequencing, plant growth promoting bacteria (PGPB), Synchytrium endobioticum

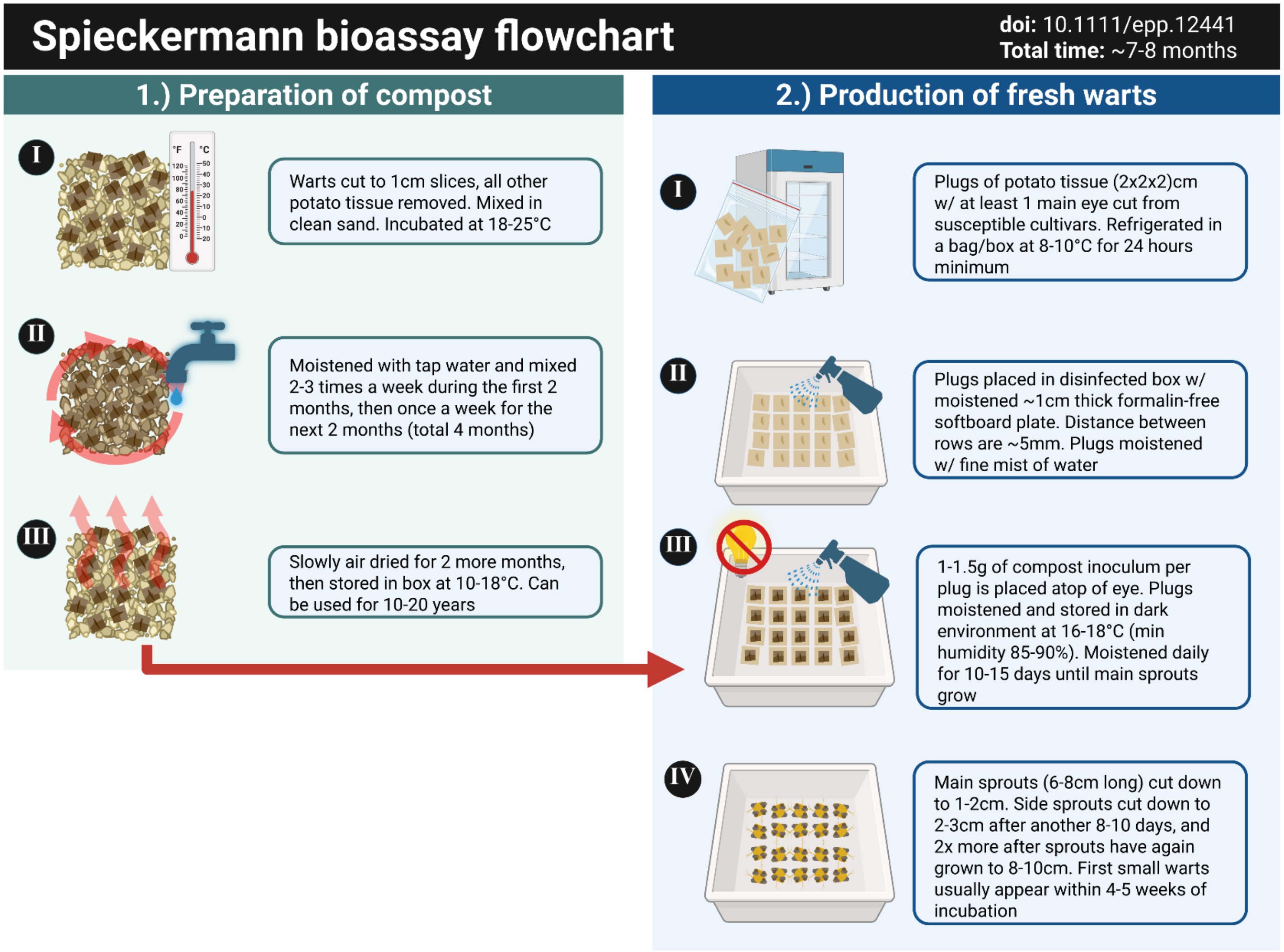

Supplementary Figure S1. Flowchart of the Spieckermann bioassay protocol, illustrating the steps used to inoculate potatoes under controlled laboratory conditions for the study of potato wart disease.

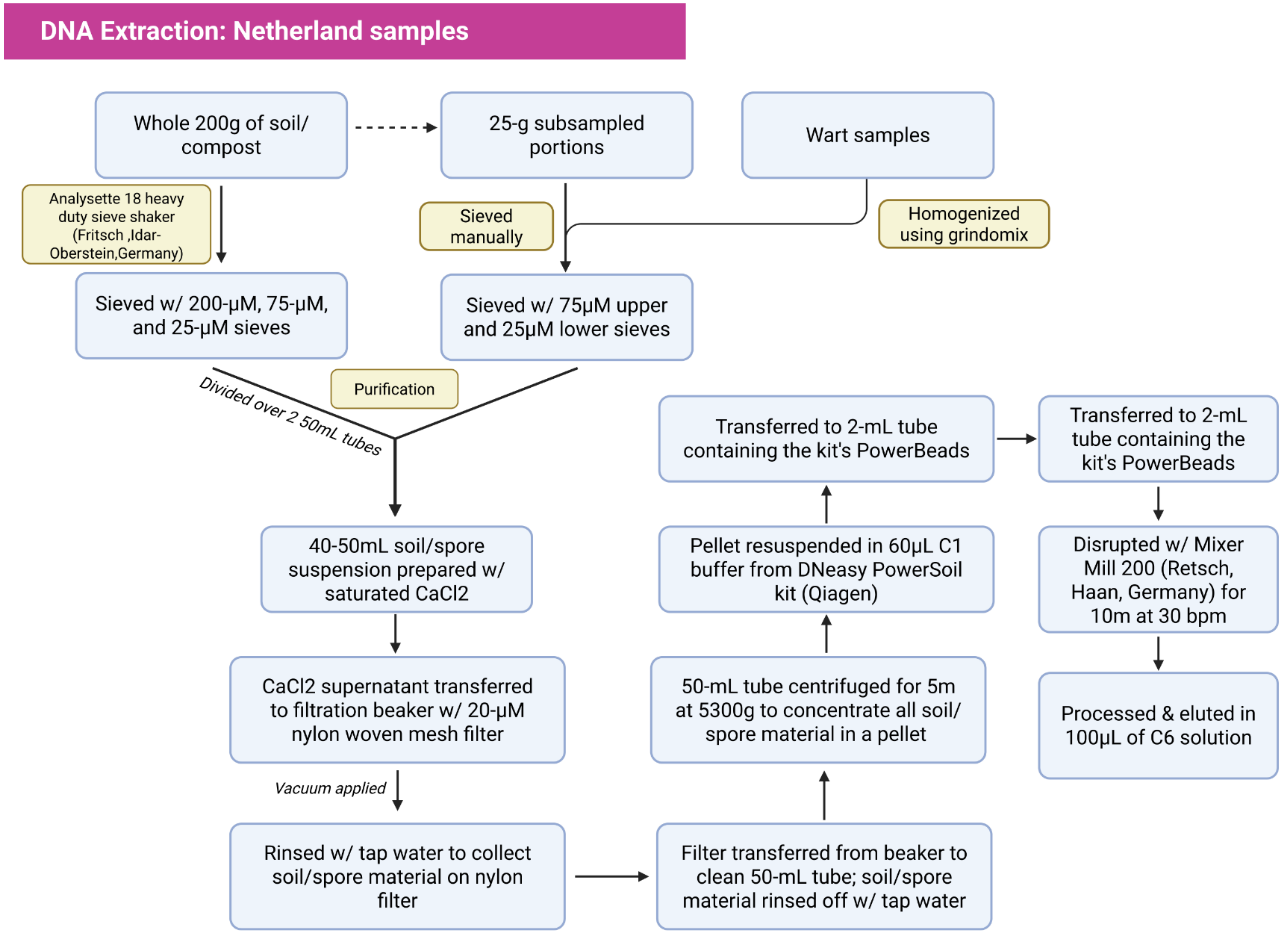

Supplementary Figure S2. Workflow summarizing sample preparation and DNA extraction procedures applied to soil and wart samples collected from the Netherlands.

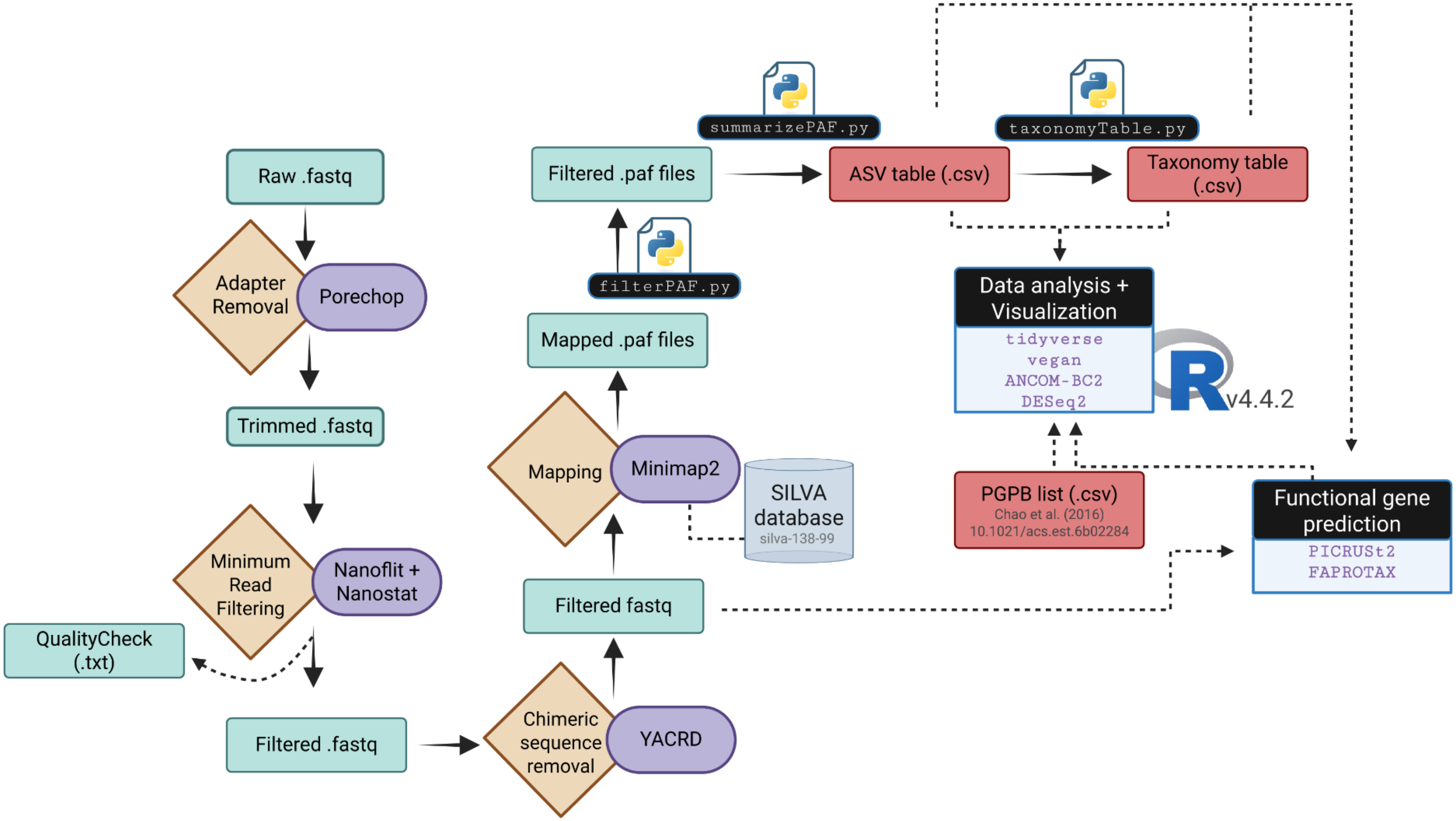

Supplementary Figure S3. Workflow illustrating the 16S rRNA gene metabarcoding data processing pipeline using Nanopore sequencing, including sequence quality control, trimming, taxonomic assignment, and downstream analyses.

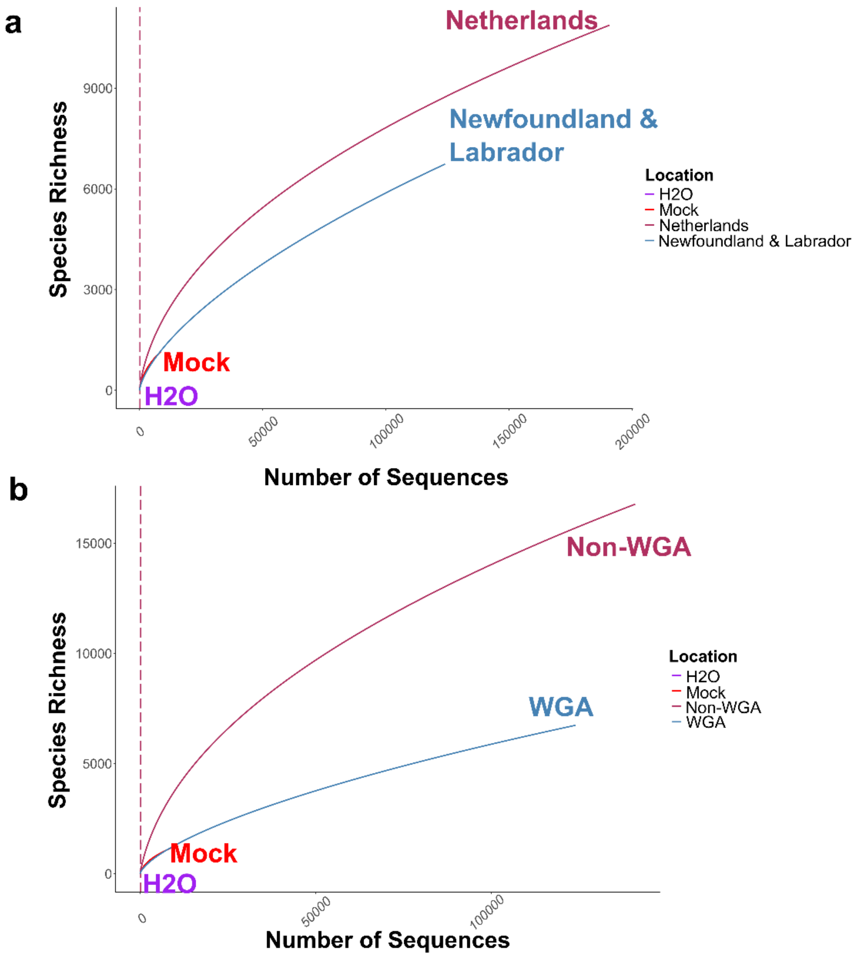

Supplementary Figure S4. Rarefaction curves showing sequencing depth for environmental DNA samples. (a) Whole genome amplified (WGA) samples from Newfoundland & Labrador and the Netherlands. (b) WGA and non-WGA samples from Newfoundland & Labrador.

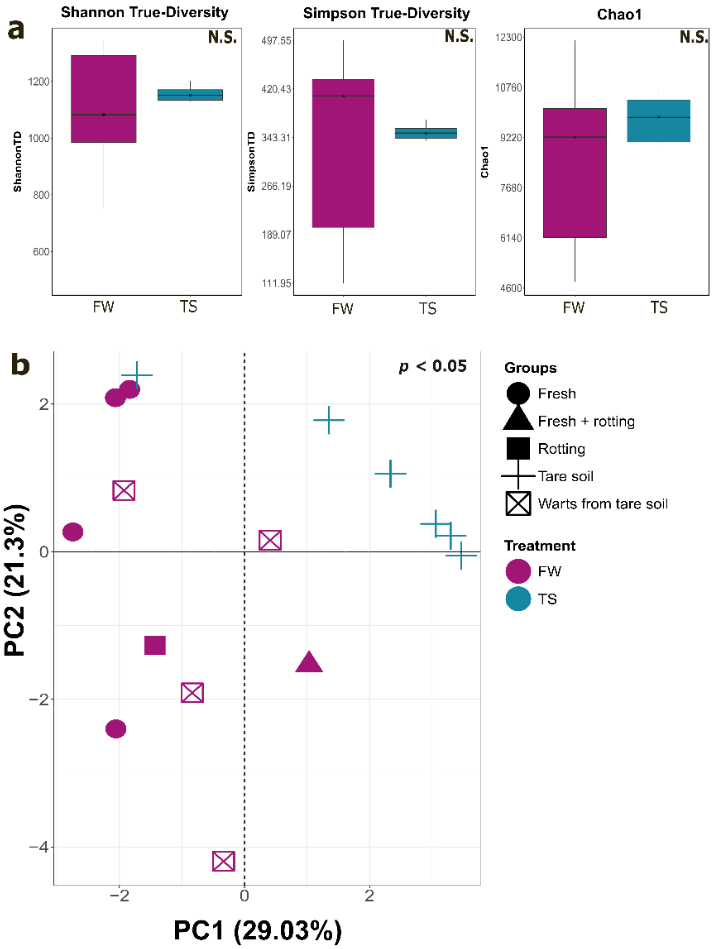

Supplementary Figure S5. Comparison of microbial communities between field wart (FW) and diseased tare soil (TS) samples. (a) Alpha diversity metrics show no significant differences between FW and TS communities ($p > 0.05$). (b) Principal component analysis (PCA) reveals significant differences in community composition between FW and TS.

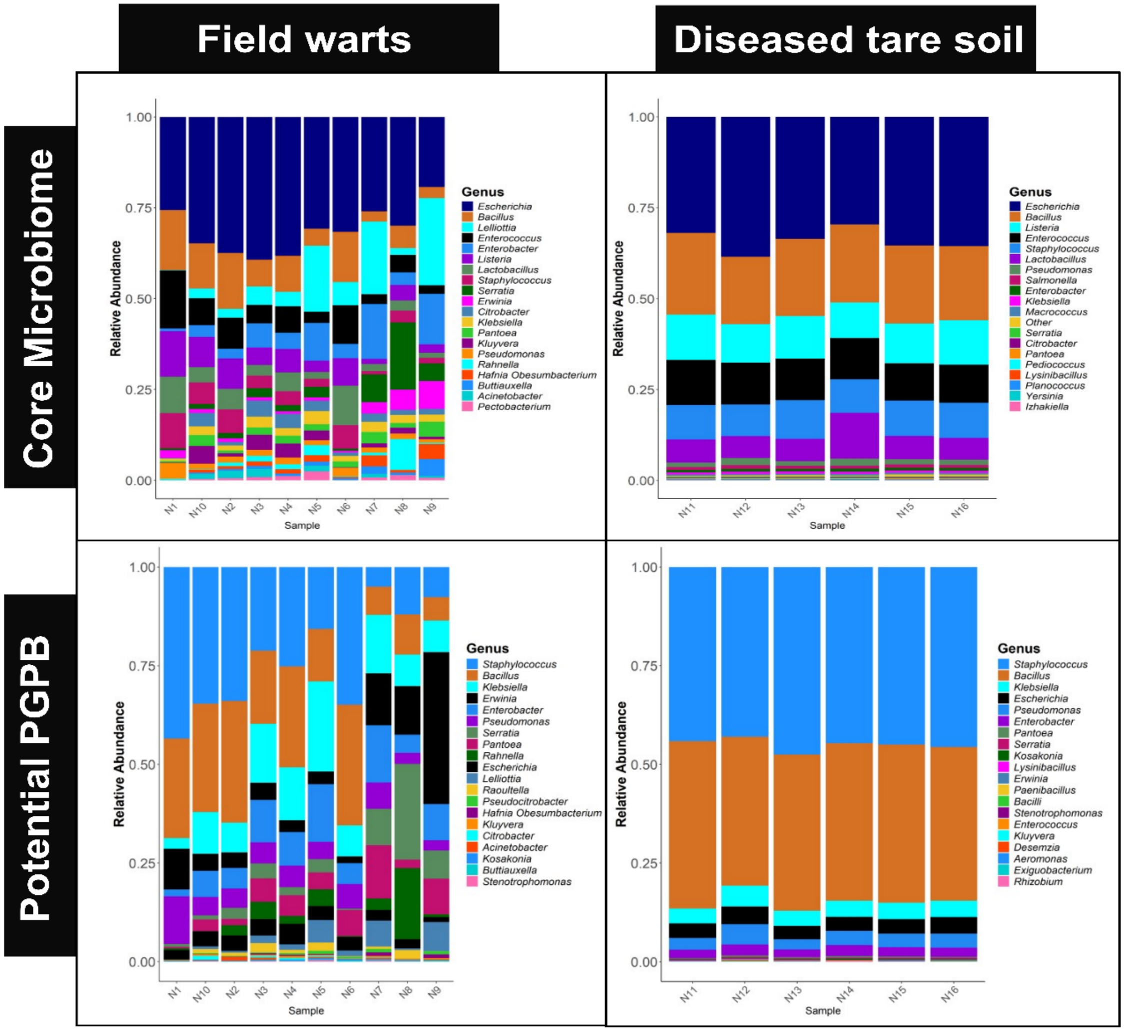

Supplementary Figure S6. Relative abundance of the 20 most abundant genera in the core microbiomes (top) and potential plant growth promoting bacterial (PGPB) communities (bottom) of field warts (left) and diseased tare soil (right).

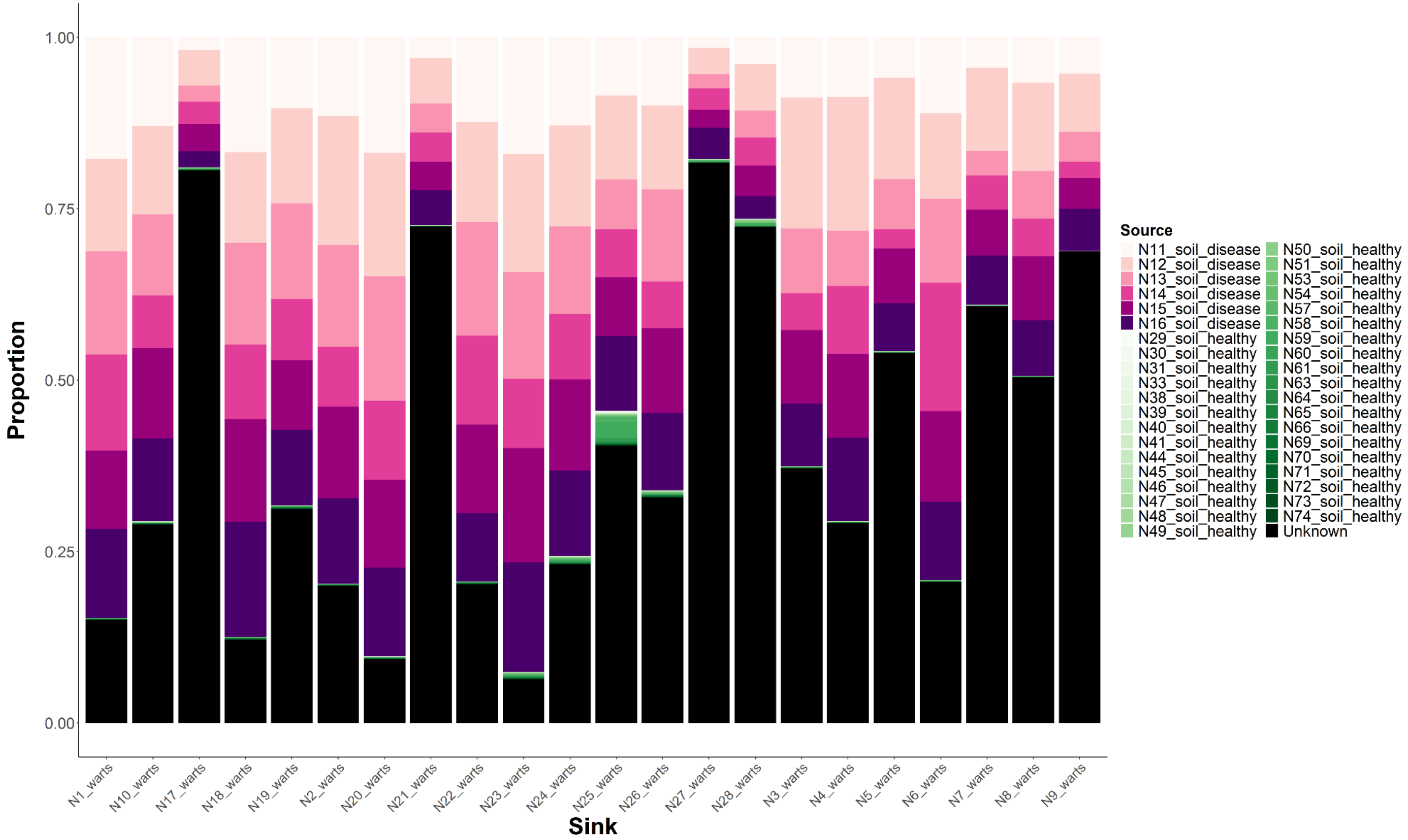

Supplementary Figure S7. Microbial source tracking using FEAST showing the estimated proportion of each wart sample's community derived from diseased soil, healthy soil, or unknown sources. Most wart communities are predicted to originate from unknown and diseased soil sources, with minimal contribution from healthy soil.

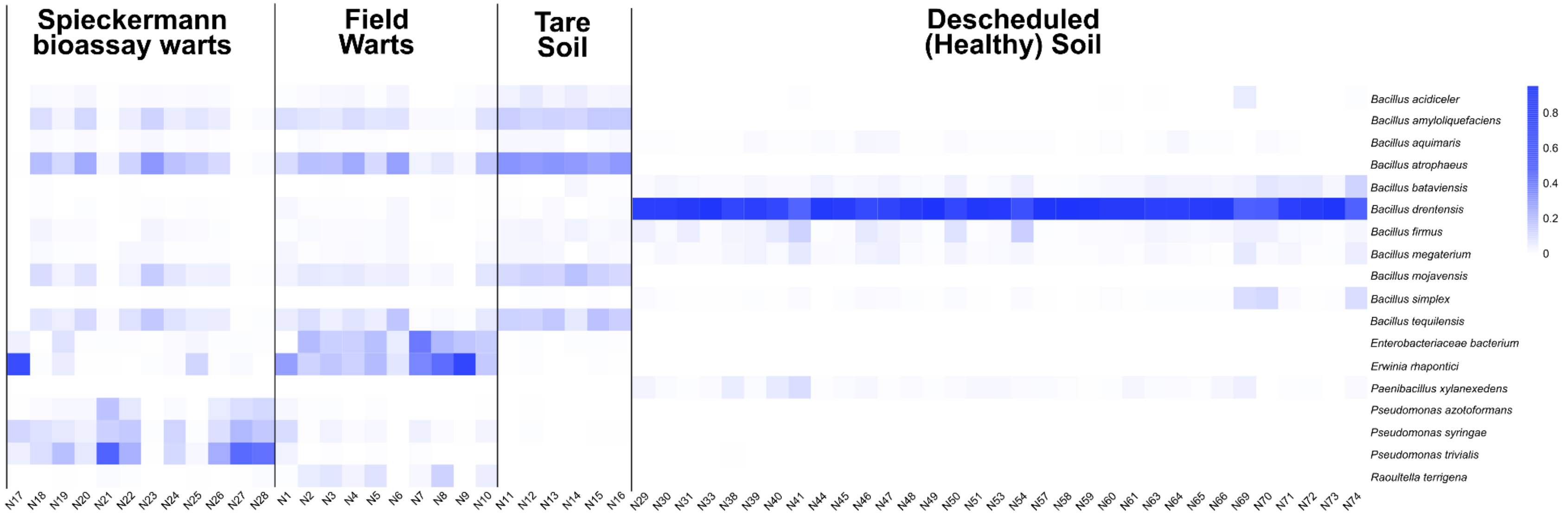

Supplementary Figure S8. Relative abundance of potential PGPB species with predicted functions relating to chitin metabolism.
